# Cold‐induced secondary dormancy and its regulatory mechanisms in *Beta vulgaris*

**DOI:** 10.1111/pce.14264

**Published:** 2022-01-28

**Authors:** James E. Hourston, Tina Steinbrecher, Jake O. Chandler, Marta Pérez, Katrin Dietrich, Veronika Turečková, Danuše Tarkowská, Miroslav Strnad, Fridtjof Weltmeier, Juliane Meinhard, Uwe Fischer, Karin Fiedler‐Wiechers, Michael Ignatz, Gerhard Leubner‐Metzger

**Affiliations:** ^1^ Department of Biological Sciences Royal Holloway University of London Egham UK; ^2^ KWS SAAT SE & Co. KGaA Einbeck Germany; ^3^ Laboratory of Growth Regulators, Palacký University and Institute of Experimental Botany Czech Academy of Sciences Olomouc Czech Republic

**Keywords:** coat dormancy, cold‐induced dormancy, embryo growth potential, endosperm weakening, germination temperature, secondary dormancy, seed transcriptomes, sugar beet

## Abstract

The dynamic behaviour of seeds in soil seed banks depends on their ability to act as sophisticated environmental sensors to adjust their sensitivity thresholds for germination by dormancy mechanisms. Here we show that prolonged incubation of sugar beet fruits at low temperature (chilling at 5°C, generally known to release seed dormancy of many species) can induce secondary nondeep physiological dormancy of an apparently nondormant crop species. The physiological and biophysical mechanisms underpinning this cold‐induced secondary dormancy include the chilling‐induced accumulation of abscisic acid in the seeds, a reduction in the embryo growth potential and a block in weakening of the endosperm covering the embryonic root. Transcriptome analysis revealed distinct gene expression patterns in the different temperature regimes and upon secondary dormancy induction and maintenance. The chilling caused reduced expression of cell wall remodelling protein genes required for embryo cell elongation growth and endosperm weakening, as well as increased expression of seed maturation genes, such as for late embryogenesis abundant proteins. A model integrating the hormonal signalling and master regulator expression with the temperature‐control of seed dormancy and maturation programmes is proposed. The revealed mechanisms of the cold‐induced secondary dormancy are important for climate‐smart agriculture and food security.

## Summary Statement

Dispersed seeds and fruits are sophisticated sensors of ambient temperature and adjust their responses to the environment via dormancy. Here we reveal the molecular and biophysical mechanisms of chilling‐induced secondary dormancy in apparently nondormant sugar beet crop seeds. Detailed knowledge of the secondary dormancy in crop species is important for climate‐smart agriculture, for breeding stress‐tolerant crops and sustainable seed technologies that mitigate the negative impacts of climate change.

## 1 INTRODUCTION

Resilience of crop establishment is vital for agricultural environments with the most vulnerable early seed and seedling stages being increasingly affected by climate change (Donohue et al., 2010; Durr & Boiffin, 1995; Fernandez‐Pascual et al., 2019; Finch‐Savage & Footitt, 2017; Walck et al., 2011). Extended growing seasons in the temperate zone and consequently the earlier sowing of crops to achieve higher yield is concomitant with an elevated risk of extreme weather events, which, in turn, threaten yields (Finch‐Savage & Bassel, 2016; Lamichhane et al., 2019; Moore & Lobell, 2015). Seeds act as environmental sensors of temperature, moisture and light to target germination to an optimal temporal window during seasons, and perhaps more importantly, to avoid germinating at the wrong time (Finch‐Savage & Footitt, 2017; Footitt et al., 2011; Walck et al., 2011). This control of germination timing is achieved by seed dormancy, which can be considered as block(s) to the completion of germination of an intact viable seed under otherwise favourable conditions, that is, after the seed becomes nondormant (Baskin & Baskin, 2014). Primary dormancy is established during seed maturation before dispersal (Chahtane et al., 2017; Finch‐Savage & Leubner‐Metzger, 2006; Graeber et al., 2012; Hilhorst, 1995; Nonogaki, 2019; North et al., 2010), whereas secondary dormancy refers to the acquisition of dormancy in a mature seed after dispersal and after the loss of primary dormancy (Finch‐Savage & Footitt, 2017; Hilhorst, 1998; Soltani et al., 2019). It is a matter of unresolved debate if some degree of primary dormancy is a necessary requirement for becoming secondary dormant. This is relevant for volunteer crop weed management (Sester et al., 2007; Soltani et al., 2019), as well as for crop seed quality and field performance (Finch‐Savage & Bassel, 2016; Ignatz et al., 2019; Kockelmann et al., 2011; Lamichhane et al., 2019).

Abiotic ambient factors, such as temperature, light and nutrient availability, during seed maturation on the mother plant, affect the degree of primary seed dormancy in *Arabidopsis thaliana* (He et al., 2014; Huang et al., 2018; MacGregor et al., 2015), and are important for sugar beet seed production (Figure S1a). Secondary dormancy can be induced in imbibed seeds of *A. thaliana* when they encounter prolonged unfavourable conditions such as darkness combined with nonoptimal temperature or limited water availability (Auge et al., 2015; Cadman et al., 2006; Finch‐Savage & Footitt, 2017). Upon entering the soil seed bank, seasonal cycling through periods of nondormancy and dormancy is central to the competitiveness of annual weed and wild species in agricultural and natural environments (Baskin & Baskin, 2006; Finch‐Savage & Footitt, 2017; Walck et al., 2005). Mature seeds of most crop species, including *Brassica napus* (oilseed rape) and *Beta vulgaris* (sugar beet), are reported to be nondormant (Hermann et al., 2007; Soltani et al., 2019). Despite the low or lacking primary dormancy of fresh mature *B. napus* seeds, the secondary dormancy potential of many oilseed rape cultivars is high, and secondary dormant seeds can remain viable in the soil for many years, which eventually can lead to the emergence of weedy volunteer *B. napus* (L. Liu, Liu, et al., 2019; Nee et al., 2015; Soltani et al., 2019). Although *B. napus* and *A. thaliana* seeds differ in primary dormancy, the induction of secondary dormancy in the two Brassicaceae relatives was associated with similar molecular mechanisms and regulation of dormancy and hormone‐related genes. In their review, Soltani et al. (2019) conclude it is likely that the capacity for induction of secondary dormancy in *B. napus* requires some degree of primary dormancy and that its induction in nondormant seeds is via a state of conditional dormancy, supporting the concept of the dormancy continuum (Baskin & Baskin, 2014; Finch‐Savage & Footitt, 2017; Finch‐Savage & Leubner‐Metzger, 2006). Beyond this, very little is known about the environmental factors and molecular mechanisms of secondary dormancy induction in crop species.

**Figure 1:**
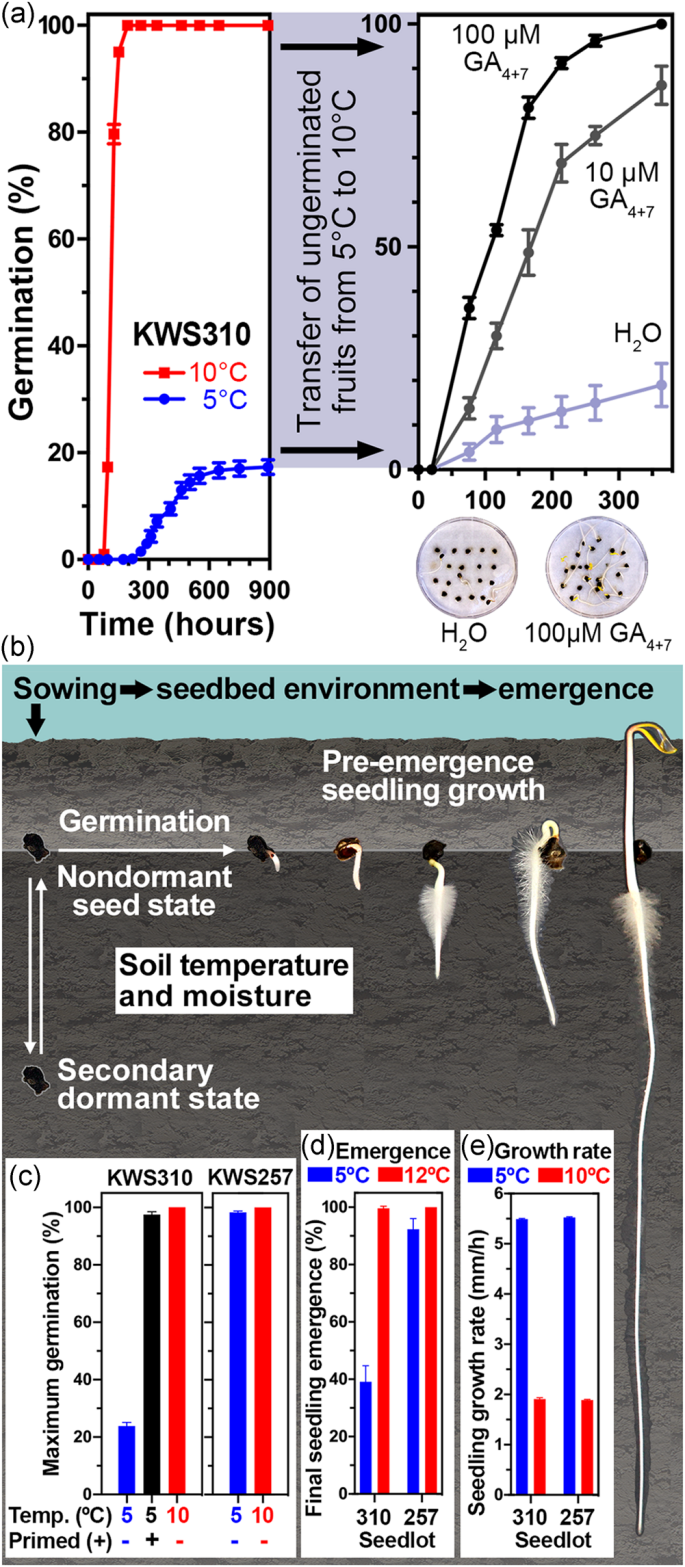
Cold‐induced secondary dormancy induction, germination and pre‐emergence seedling growth of *Beta vulgaris*. (a) Cold‐induced secondary dormancy induction of seedlot KWS310. Germination of fruits at 5°C and 10°C (*left panel*; *n* = 5 plastic boxes of 100 fruits). Ungerminated fruits from the 5°C treatment were transferred to 10°C and imbibed in either H_2_O (control) or in the presence of gibberellin (GA_4+7_; *right panel*; *n* = 4 Petri dishes). The secondary dormancy of the ungerminated fruits in the control series was physiological dormancy as evident from its release by GA treatment. (b) Relation between stages of secondary dormancy, germination and pre‐emergence seedling growth. (c) Effect of seed priming and temperatures on the maximum germination percentages of contrasting sugar beet lots (for details see Figures S2b and S5b). (d) Final seedling emergence from the soil at two temperatures. (e) Seedling pre‐emergence growth rates derived from the slopes in Figure S4b. Mean values ± SEM are presented [Color figure can be viewed at wileyonlinelibrary.com]

Sugar beet, *B. vulgaris* subsp. *vulgaris* var. *altissima* Doell. (Amaranthaceae), is a recently domesticated crop plant species and differs in its genome considerably from *A. thaliana*, the *Brassica* crops and other crop species (Dohm et al., 2014). Sugar beet accounts for about a third of the world's sugar production and is the major source of sugar in temperate zones. Sugar beet is a typical example of a spring crop with major climate change impacts due to altered growing‐season temperature and precipitation regimes (Durr & Boiffin, 1995; Lamichhane et al., 2019; Moore & Lobell, 2015). While extending the growing season by earlier sowing contributes to increased sugar yield, it also comes with the increased risk of spring frosts (Figure S1), which may damage the early seed and seedling stages and thereby crop establishment (Deihimfard et al., 2019). The sugar beet dispersal unit is a nondormant fruit, consisting of the true seed encased by the pericarp (fruit coat) (Hermann et al., 2007; Ignatz et al., 2019; Kockelmann et al., 2011). The true seed is composed of a coiled embryo that surrounds the central perisperm, covered by a thin seed coat and an endosperm covering the radicle (embryonic root). In general, germination is a complex physiological process wherein, once all dormancy blocks have been removed, expansion growth of the embryo culminates in rupture of the covering layers and emergence of the radicle (Finch‐Savage & Leubner‐Metzger, 2006; Nonogaki, 2006; Steinbrecher & Leubner‐Metzger, 2017). The completion of germination by radicle emergence is succeeded by pre‐emergence seedling growth (Figure 1), a distinct developmental process that is also affected by soil temperature and moisture.

Here we investigated the molecular and biophysical mechanisms of cold‐induced secondary dormancy. While the role of heat in plant dormancy has been studied, that chilling temperatures can induce secondary seed dormancy is a phenomenon for which almost nothing is known. Detailed knowledge of secondary dormancy mechanisms in crop species with apparently nondormant seed is important for climate‐smart agriculture and food security.

## 2 MATERIALS AND METHODS

### 2.1 Plant material

Fruits of sugar beet (*B. vulgaris* L. subsp. *vulgaris* var*. altissima* Doell. *L*.) seedlots KWS310 and KWS257 were obtained from KWS SAAT SE & Co. KGaA (Einbeck). Fruits were produced in Italy in 2014 (Figure S1) according to the commercial practice (Kockelmann et al., 2011). At harvest, plants were cut and left on swath for drying. After threshing, if required, additional drying occurred. Harvested fruits were cleaned, calibrated into various size classes and polished to remove part of the pericarp. Before use, fruits were additionally washed and re‐dried to appropriate moisture content. Both polishing and washing aim to reduce the amount of putative germination inhibitors present in the pericarp of the sugar beet fruit (Ignatz et al., 2019). Mean moisture content was determined to be 9.75% (dry weight) as the reduction of five sets of 15 fruits following a drying process of 8 h at 105°C.

### 2.2 Physiological and biomechanical methods

To score germination over time white plastic boxes (180 × 135 × 65 mm) with transparent lids containing pleated filter paper (Hahnemuehle) were used, each box containing 100 sugar beet fruits and 30 ml dH_2_O (Hermann et al., 2007). Sets of three boxes were incubated at 5 or 10°C in darkness (incubator MIR‐254‐PE; Panasonic). The completion of germination was defined as the radicle protruding through and beyond the margin of the operculum of the sugar beet fruit. About 80% of KWS310 fruits imbibed at the 5°C temperature remained ungerminated even when incubated for >700 h at 5°C (Figure 1). A subset of these fruits was transferred to 9 cm Petri dishes (*n* = 4 × 20 fruits) and incubated further at 10°C but under three different regimes: 4 ml H_2_O (control), 4 ml of 10 µM or 4 ml of 100 µM gibberellin (GA_4+7_, Duchefa Biochemie B.V) and germination was assessed again (Figure 1a). For the biomechanical analyses, sugar beet fruits were imbibed in white plastic boxes as previously described (Hermann et al., 2007) for indicated time and temperature [Control 12 h (5°C and 10°C), *T*_1%_ (255 h for 55°C, 85 h for 10 °C), *T*_50%_ (120 h for 10°C), *T*_SD_ (600 h for 5°C), *T*_SD + 10 d_ 10°C (600 h at 5°C + 240 h at 10°C)]. To assess the biomechanical properties of isolated sugar beet endosperm the seed was removed from the pericarp, dissected and testa and radicle removed. The intact micropylar endosperm tissue was glued with Loctite Super Glue Power Flex Gel (Henkel) to a metal sample holder (hole size, 0.65 mm). A metal probe with a diameter of 0.3 mm was driven into the sample at a speed of 1 mm/min while recording force and displacement (Zwick Roell ZwickiLine Z0.5). The puncture force (tissue resistance) was determined to be the maximal force from the force‐displacement curves (Graeber et al., 2014; Holloway et al., 2021; Steinbrecher & Leubner‐Metzger, 2017). Quantifying the embryo growth potential and pre‐emergence seedling growth was performed as described in Supporting Information Methods.

### 2.3 Biochemical methods and transcriptome analyses

**Figure 2:**
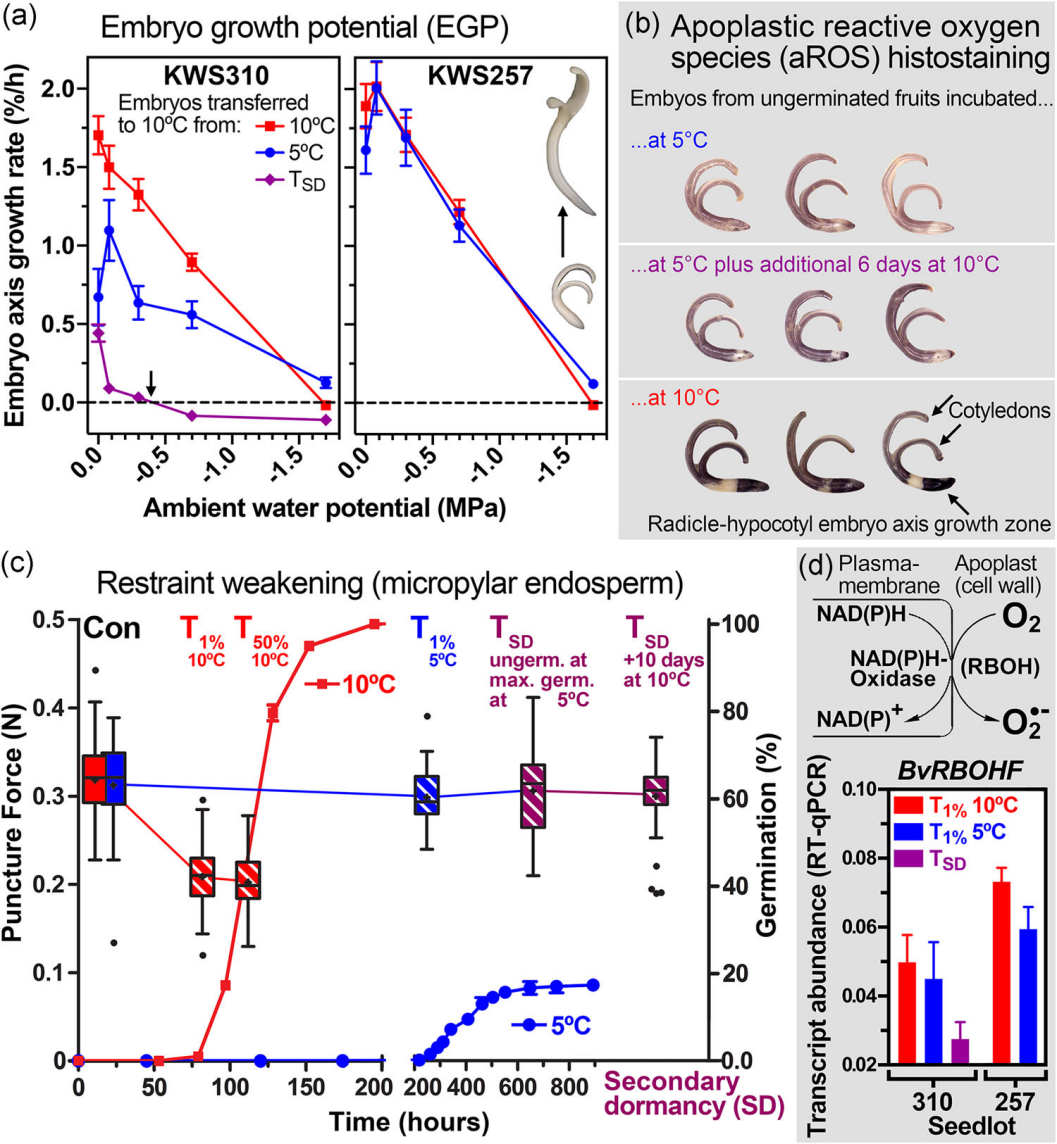
Inhibition of embryo growth and endosperm weakening associated with the cold‐induced secondary dormancy of sugar beet. (a) Embryo growth potential (EGP) analysed as embryo axis elongation growth of isolated embryos under different conditions of temperature and water potential (*n* ≥ 13). Embryos were extracted from KWS310 fruits either at 5°C or 10°C at the respective *T*_1%_ or from secondary dormant fruits (*T*_SD_) and incubated at the indicated water potential (see Figure S5 for details). Note that the growth potential of embryos from secondary dormant fruits was severely reduced (arrow marks threshold water potential where growth ceases). The contrasting lot KWS257 (Figure S2) served as comparison. (b) Histochemical analysis of apoplastic reactive oxygen (aROS) using the NBT histostain for superoxide with embryos extracted from KWS310 fruits imbibed in different temperature regimes. Compare the intense aROS histostain in the growing zone (hypocotyl‐radical axis) of 10°C embryos with 5°C and upon secondary dormancy. (c) Biomechanical analysis of KWS310 micropylar endosperm weakening. Puncture force (tissue resistance) of micropylar endosperm tissue of sugar beet fruits imbibed at the two temperature regimes (5 vs. 10°C) and during secondary dormancy (*T*_SD_); the overlay shows the germination kinetics. Con, control early timepoint; *T*_1%_, physiological timepoints at which 1% of the population has germinated; *T*_50%_, the timepoint at which half the population has germinated; *T*_SD_ (ungerminated fruits analysed at maximum germination at 5°C), timepoint at which cold‐induced secondary dormancy has been induced during the imbibition at 5°C; *T*_SD_ (+10 days at 10°C), timepoint during secondary dormancy maintenance for the subpopulation of ungerminated fruits, which has been transferred from 5°C for 10 days incubation at 10°C. Note that a rapid decrease in endosperm tissue resistance (puncture force) occurred before the completion of germination at 10°C, whereas this endosperm weakening was blocked at 5°C and during secondary dormancy fruits. Mean values, medians and whisker box plots are presented, *n* ≥ 29. (d) Transcript abundances (RT‐qPCR) of the aROS producing NAD(P)H oxidase *BvRBOHF* in KWS310 and KWS257 sugar beet seeds at their respective *T*_1%_ timepoints at 5°C (blue), 10°C (red) and *T*_SD_ (purple); for a detailed *BvRBOHF* analysis, see Figure S8. Mean values ± SEM are presented [Color figure can be viewed at wileyonlinelibrary.com]

**Figure 3:**
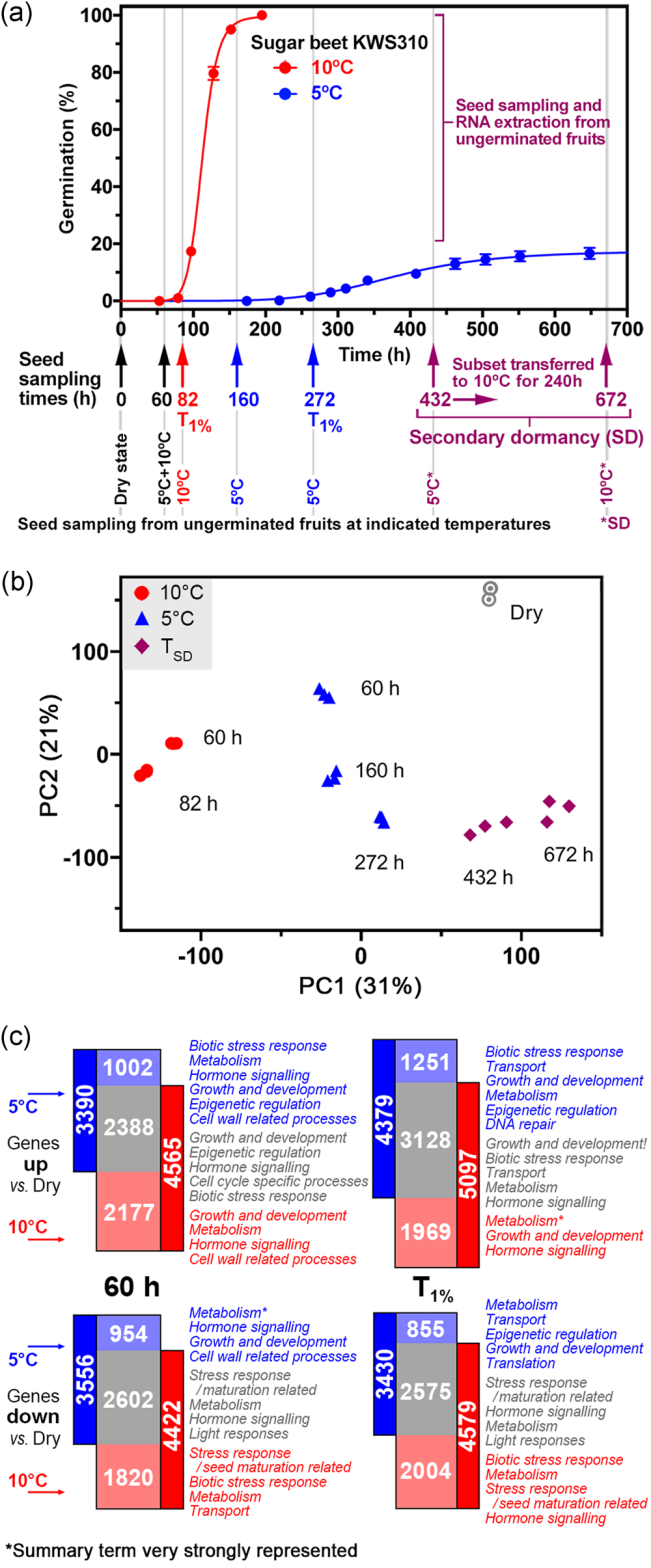
Transcriptome analysis of cold‐induced secondary dormancy. (a) The sampling scheme for RNA extraction from sugar beet KWS310 seeds. Seed samples were taken from dry fruits (0 h) and fruits imbibed at 10°C for 60 and 82 (*T*_1%_) hours. Seed samples were taken from fruits imbibed at 5°C for 60, 160, 272 (*T*_1%_) and 432 h. A subset of ungerminated fruits incubated at 5°C for 432 h was transferred to 10°C for a further 240 h and then sampled at 672 h. The time taken for 1% of the population of fruits to complete germination (*T*_1%_) was determined using germinator software (Joosen et al., 2010). Note that secondary dormancy (SD) was induced by the cold treatment (5°C) as indicated in the graph. (b) Principal component analysis (PCA) of global gene expression of sugar beet KWS310 samples confirms that variability between replicates is low and that the different imbibition temperatures and timepoints were clearly distinguishable. (c) An overview of the number of differentially expressed genes (DEGs) at both the physical time point (60 h; left panel) and the physiological timepoint (*T*_1%_; right panel) compared to dry seed transcriptome. The number of DEGs up or down versus dry seeds are indicated within the plots as well as the overlap represented in both groups. Summary terms that are common in each category are noted next to the relevant bar; * indicates that in this case, metabolism is strongly represented, significantly more than other summary terms. See Figure S7 and Supporting Information Data S2 for additional DEGs comparisons and details on the GO term analysis [Color figure can be viewed at wileyonlinelibrary.com]

Histochemical staining for apoplastic reactive oxygen species (aROS) to visualize apoplastic superoxide (Figure 2b) and phytohormone quantification by UPLC‐ESI (+)‐MS/MS were performed as described in Supporting Information Methods. For the transcriptome analyses, true seeds (4 × 100) extracted from KWS310 fruits were sampled when dry, following imbibition at the time points specified (Figure 3a). RNA was extracted and RNA‐seq conducted with three biological replicate RNA samples as described in detail in the Supporting Methods. Genes with a log2FC >1 and adjusted *p* < 0.05 or genes with log2FC < −1 and adjusted *p* < 0.05 were considered as differentially expressed genes (DEGs). BeetSet‐2 Gene models (Minoche et al., 2015) were annotated with Gene Ontology (GO) terms, best BLAST hits in UniProt database using Pedant (Biomax Informatics AG) (Riley et al., 2007) (Supporting Information Data S1). PCA was performed in R using prcomp (centre and scale = TRUE) after filtering unexpressed genes (sum expression across samples = 0). GO term enrichment in lists of DEGs (DEGs in pairwise comparisons between two samples, or DEGs common or unique across multiple pairwise comparisons) was analysed with the topGO Bioconductor package (version 2.34.0) using the classic method and fisher test (Alexa & Rahnenfuhere, 2018). To summarize biological process (BP) GO terms, all GO terms that belonged to the top 100 GO terms in any of the outputs were assigned a simplified category. The abundance of the simplified categories in the top 100 GO terms of each output was used to annotate Venn diagrams (Figures 3, S7 and Data S2). K‐means clustering for XTHs was generated using Morpheus (July 2, 2019) with (Euclidean distance, maximum 1000 iterations) of genes based on *Z*‐score normalized gene expression. Spaghetti plots were produced in *R* for all genes in a list, and mean expression using ggplot2 (Wickham, 2016). Genes belonging to groups that were averaged in the main text plots are shown in Table S1. Detailed information on how to use the Gene Expression Viewer is provided in Supporting Instructions. For conducting the reverse‐transcription quantitative polymerase chain reaction (RT‐qPCR), 1 μg DNase I‐treated RNA was used for cDNA synthesis with random hexamer primers, using the Invitrogen™ SuperScript™ III First‐Strand Synthesis System (Thermo Fisher Scientific). RT‐qPCR reactions were performed in a CFX96 Touch™ Real‐Time PCR Detection System (Bio‐Rad), using ABsolute qPCR SYBR Green Mix (Thermo Fisher Scientific) and primer pairs listed in Table S2, with the following parameters: 95°C for 15 min, 40 cycles with 95°C for 15 s, and 60°C for 30 s, and 72°C for 30 s, then 65°C for 31 s. Melt‐curve analysis verified the absence of primer‐dimer artefacts and amplification of a single product from each qPCR assay. PCR efficiencies and *Cq* values were calculated using Real‐time PCR Miner algorithm using raw fluorescence data as input (Graeber et al., 2011). The geometric mean of *B. vulgaris BvVHA‐A3* and *BvMGL* genes (Table S2) was used as a reference for normalisation. All RT‐qPCR experiments were performed using three independent biological replicates.

## 3 RESULTS

### 3.1 Low imbibition temperatures induce secondary dormancy in sugar beet fruits

Low temperature incubation (chilling at 5°C) of imbibed *B. vulgaris L*. fruits from the lot KWS310 was found to induce secondary dormancy (Figure 1). A screen of 19 sugar beet lots for their germination responses at 5°C identified KWS310 as highly suited for an exemplified analysis whereas the other sugar beet lots, including KWS257, which shares the same production site and year with KWS310 (Figure S1), did not exhibit this phenomenon (Figure S2). The maximal germination of KW310 fruits imbibed at 5°C was only ~18% at >600 h compared to 100% at 160 h when imbibed at 10°C (Figure 1a, left panel). Simply transferring the >80% ungerminated fruits from 5°C to the more optimal conditions (10°C) did not restore germination, as >80% of these fruits did not germinate at 10°C (Figure 1a, right panel). We, therefore, concluded that the prolonged imbibition at low (5°C) temperature may have induced secondary dormancy in the apparently nondormant crop seedlot. Figure 1c shows that sugar beet seed priming prevented this for KWS310 consistent with the known positive effects of the priming treatment on seedling emergence in general (Ignatz et al., 2019; Kockelmann et al., 2011; Salimi & Boelt, 2019). Figure S3 further demonstrates that imbibition in the presence of exogenous gibberellin (GA_4+7_) or scarification of the pericarp (by removal of the operculum) prevented the secondary dormancy induction. Figure 1a shows that treatment of the ungerminated KWS310 fruits from the prolonged incubation at 5°C with exogenous GA_4+7_ resulted in 100% germination, which confirms their viability and that the secondary dormancy induced by the chilling treatment is a nondeep physiological dormancy according to the widely accepted classification system of Baskin and Baskin (2014). Release of the secondary dormancy by GA_4+7_ treatment was also possible at 5°C (Figure S3e). To further investigate the observed phenomenon beyond germination, we compared the seedling growth of KWS310 and KWS257 upon chilling.

To test if these chilling responses are relevant for early spring sowing of sugar beet (Figure S1b), we also compared seedling emergence from the soil at 5°C. Seedling emergence was indeed reduced in KWS310 upon chilling, but not affected in KWS257, which exhibited full emergence (Figure 1d) and full germination (Figure S2b). The observed reduced seedling emergence of KWS310 at 5°C mimicked early season frost and chilling temperatures (Figure S1b), which therefore may not only damage the vulnerable early seedling stages (Deihimfard et al., 2019) but may also reduce seedling emergence due to secondary dormancy induction in the field (Figure 1b). To investigate sugar beet pre‐emergence seedling growth independently from the germination process (Figure 1b), we developed a quantitative assay (Figure S4a). The comparative analysis of KWS310 and KWS257 demonstrated that pre‐emergence seedling growth did not appreciably differ between the two lots (Figure S4b). While the quantified growth rates differed between the temperature regimes, they did not differ between KWS310 and KWS257 (Figure 1e). We, therefore, conclude that the reduced seedling emergence of KWS310 (Figure 1d) is not caused by reduced pre‐emergence seedling growth, but by chilling‐induced secondary dormancy (Figure 1b). The focus of our subsequent experiments was therefore on investigating the underpinning mechanisms of the chilling‐induced switch between germination and secondary dormancy.

### 3.2 The chilling‐induced secondary dormancy is associated with reduced embryo growth potential combined with blocked endosperm weakening

The completion of germination by radicle emergence depends on the balance of the opposing forces between the embryo growth potential (EGP) and the restraint of the seed‐covering layers (endosperm, seed coat, fruit coat) (Figure S5a, Steinbrecher & Leubner‐Metzger, 2017). Cell‐wall loosening is required for irreversible cell expansion by water uptake as the force that drives embryonic axis elongation. To investigate if the EGP was reduced by imbibition at low (5°C) temperature and secondary dormancy as compared to 10°C, we conducted physiological, histochemical and molecular analyses. To study this, embryo growth data was acquired for KWS310 and KWS257 at comparable physical and physiological times (*T*_1%_, i.e., the onset of germination completion in the fruit populations) and after secondary dormancy (Figures 2, S5b and S6). Incubation at 5°C reduced subsequent embryo growth of KWS310 at different ambient water potentials (Ψ) at 10°C substantially, and this was especially evident for embryos from secondary dormant fruits (Figure 2a). Elongation of embryos from secondary dormant fruits was reduced to almost zero when Ψ was only slightly reduced to −0.1 MPa, and at further reduced ambient Ψ (−0.7 MPa) tissues were effectively shrinking in size or too degraded to be measured (−1.7 MPa).

In contrast to KWS310, the EGP of KWS257 was not reduced by the chilling treatment (Figure 2a). The comparative analysis of embryo growth further revealed that seed priming increased the EGP of both lots (Figure S5b), that embryo axis growth of early isolated embryos occurred at a lower rate at both 5°C and 10°C (Figure S6a) and that the embryo axis (EA; radicle and lower hypocotyl) is the major embryo elongation zone and also the main target for the reduction in EGP in lot KWS310 (Figure S6b). Consistent with the reduced EGP, embryos from fruits imbibed at 5°C also exhibited severely reduced production of aROS in the cell walls of the embryo elongation zone, that is, the lower hypocotyl and radicle, and also in the cotyledons (Figure 2b). Biochemically, the aROS production in the cell walls is achieved by the enzymatic activity of NADPH oxidase/respiratory burst oxidase homolog (RBOH) proteins (Figure 2d). RBOH and other cell wall remodelling proteins provide a biochemical mechanism for cell wall loosening in the embryo elongation zone required for cell expansion driven by water uptake into the vacuole (Müller et al., 2009; Steinbrecher & Leubner‐Metzger, 2017). Consistent with the higher EGP of KWS257 compared to KWS310 (Figure 2a) and the role of aROS production in embryo growth, *BvRBOHF* gene expression was higher in KWS257 compared to KWS310 and further reduced in KWS310 upon secondary dormancy (Figure 2d).

In mature seeds of many species, the endosperm is a mediator of communication between the embryo and the ambient environment (Chahtane et al., 2017; Finch‐Savage & Leubner‐Metzger, 2006; Yan et al., 2014). It is a major source of abscisic acid (ABA) production to inhibit embryo expansion growth and to confer coat‐dormancy by inhibiting endosperm weakening (Graeber et al., 2014; Müller et al., 2009; Steinbrecher & Leubner‐Metzger, 2017). Endosperm weakening is often a prerequisite for the completion of germination and is environmentally regulated via hormonal networks and the action of cell wall remodelling proteins. To determine biomechanical changes on a mechanistic level in response to low temperature the tissue resistances of the sugar beet micropylar endosperms were investigated using puncture force analysis (Figures 2c and S5a). The micropylar endosperms from fruits incubated at 10°C showed a ~40% reduction in tissue resistance already at *T*_1%_, with no further tissue weakening observed at later timepoints. In contrast to this, during the imbibition of sugar beet fruits at 5°C no reduction in tissue resistance was observed until the end of the 432‐h period, and hence the cold temperature has blocked the endosperm weakening (Figure 2c). Interestingly, transfer of secondary dormant fruits to 10°C did not result in any changes in tissue resistance, the endosperm weakening remained blocked (Figure 2c). This suggests that the biochemical processes that were initiated during early germination and subsequently downregulated by the chilling treatment did not cause changes in the physical properties of the micropylar endosperm. Endosperm weakening, therefore, seems to be a pre‐requisite for sugar beet germination and is blocked during imbibition at 5°C. It is also blocked during secondary dormancy, which seems to constitute a mechanism causing the secondary dormancy when combined with the reduced EGP.

### 3.3 Transcriptome analysis reveals distinct gene expression in different temperature regimes and upon secondary dormancy induction and maintenance

To investigate the underpinning molecular mechanisms of the cold‐induced secondary dormancy, we conducted a transcriptome analysis via RNAseq. Sugar beet fruits of lot KWS310 were incubated at 5°C and 10°C and true seeds were sampled at several key physical and physiological timepoints (Figure 3a): Dry state, for the 10°C imbibed samples at 60 and 82 h (*T*_1%_), for the 5°C imbibed samples at 60, 160 and 272 h (*T*_1%_). Seeds from ungerminated secondary dormant fruits imbibed at 5°C were sampled at 432 h (induction phase) and a subset of these fruits was transferred from 5°C to 10°C (at the 432 h timepoint). These transferred secondary dormant fruits were also sampled after a further 10‐day incubation at 10°C, that is, at the 672 h sampling point (Figure 3a). RNAseq of RNA extracted from triplicate samples of true seeds and subsequent RNAseq analyses were conducted. Figure 3b shows the global transcript expression patterns following principal component analysis (PCA). In this PCA, the secondary dormant samples cluster together, separated from the earlier sampling points. For the analysed transcriptomes, the eigenvector with the biggest influence explained 31% of the variance in the first component (PC1), which separated the two imbibition temperatures as well as the induction/maintenance of secondary dormancy within the 5°C regime. The second component (PC2) explained 21% of the variance and separated the data by imbibition time until secondary dormancy was induced.

Analysis of differentially expressed genes (DEGs) relative to the dry state revealed that at 60 h (Figure 3c, left panels) for the low‐temperature regime (5°C) 3390 genes were upregulated compared to the dry seed of which 1002 were exclusively upregulated by the imbibition at 5°C. For the 10°C temperature regime more than twice as many genes (2177) were exclusively upregulated (of the total 4565 genes). At *T*_1%_ (Figure 3c, right panels) a similar picture emerged with a higher number of upregulated genes overall: 1251 genes exclusively upregulated at 5°C, 1969 exclusively upregulated at 10°C and 3128 upregulated by both temperatures. Similar numbers of genes were downregulated at 60 h and at *T*_1%_ (Figure 3c). Gene Ontology (GO) term enrichment analysis is presented next to these DEG lists. Together with further comparisons of DEGs presented in Figure S7, the results collectively indicate that the main changes caused by low temperature had already occurred within the first 60 h of imbibition and then eventually led to secondary dormancy induction by the chilling treatment at 5°C. Lists of these DEGs and details on the GO term analysis for the various comparisons are presented in Supporting Information Data Sets S1 and S2. The transcriptome results are accessible via the Gene Expression Viewer (Supporting Information for Gene Expression Viewer File and Detail Instructions on how to use it). The induction and maintenance of secondary dormancy were mediated by transcriptome changes in strongly represented gene categories involved in stress responses, hormone signalling, growth development, metabolism and cell wall‐related processes in sugar beet fruits (Figures 3c and S7; Supporting Information Data S2). We, therefore, analysed the hormone contents and transcript expression patterns, which revealed molecular mechanisms underpinning the cold‐induced secondary dormancy in the apparently nondormant crop seeds.

### 3.4 The role of the hormonal network and cold signalling pathways in the cold‐induced secondary dormancy of sugar beet

**Figure 4:**
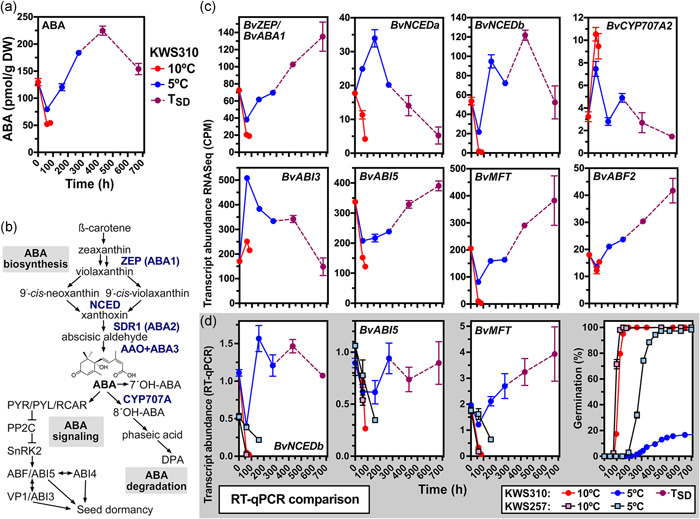
Abscisic acid (ABA) contents, and regulation of ABA‐ and ethylene‐related genes implicated in the cold‐induced secondary dormancy. (a) ABA contents in sugar beet seeds extracted from fruits incubated at the indicated temperature regimes (*n* = 5 relicates). (b) ABA metabolism and signalling. (c) Transcript abundances of ABA‐ and ethylene‐related genes in sugar beet seeds incubated at the indicated temperature regimes: The red line represents fruits incubated at 10°C, the blue line incubated at 5°C and the transition to purple represents secondary dormancy induction and maintenance (*T*_SD_, 5°C until 432 h and subsequent incubation at 10°C). Results from the transcriptome analysis of KWS310: CPM, counts per million mapped reads of *n* = 3 RNA samples (biological triplicates each from 100 sugar beet seeds); see Figure 3a for detail sampling scheme and Supporting Table S1 for the *Beta vulgaris* gene names and IDs. (d) RT‐qPCR analysis of transcript abundances of the contrasting KWS310 and KWS257 seedlots (*n* = 3 RNA samples). For primer sequences and reference genes see Supporting Table S1; note the contrasting germination patterns of these seedlots. See main text for gene abbreviations. Mean values ± SEM are presented. RT‐qPCR, reverse‐transcription quantitative polymerase chain reaction [Color figure can be viewed at wileyonlinelibrary.com]

The hormone ABA, known to induce and maintain seed dormancy of many species (Chahtane et al., 2017; Finch‐Savage & Leubner‐Metzger, 2006; Yan et al., 2014), was quantified in seeds extracted from dry and imbibed sugar beet fruits and the contents were compared with the expression pattern of ABA‐related genes (Figure 4). The endogenous ABA content rapidly decreased in seeds imbibed at 10°C, while at 5°C it only slightly decreased initially until 60 h. The ABA content subsequently increased ~3‐fold at 5°C and during secondary dormancy induction and remained at a high level in secondary dormant seeds (Figure 4a). Patterns in ABA content across two temperature regimes suggested that gene expression of key ABA‐related genes (Figure 4b) would differ accordingly. Conforming to this the *B. vulgaris* key ABA biosynthesis genes for the zeaxanthin epoxidase *BvZEP (BvABA1)* and 9‐*cis*‐epoxycarotenoid dioxygenase *BvNCED* were downregulated at 10°C and upregulated at 5°C before the secondary dormancy induction (Figure 4c); the same finding was made for *BvABA3* (Figure S8b). The *BvZEP* transcript abundances further increased during secondary dormancy maintenance. This finding was verified by RT‐qPCR analysis of *BvNCEDb* expression, which confirmed the upregulation at 5°C in KWS310 and demonstrated that in the contrasting seedlot, KWS257 *BvNCEDb* is as expected downregulated at 5°C (Figure 4d). Additionally, transcript abundances of ABA 8′‐hydroxylase (*BvCYP707A*), the enzyme initiating ABA degradation, were upregulated at 10°C and downregulated at 5°C during the induction and maintenance of secondary dormancy in KWS310 (Figure 4c).

Several ABA‐related dormancy and maturation regulators are involved in ABA signalling (Graeber et al., 2012; Leprince et al., 2017; Yan et al., 2014) and chilling (Figure 4b). The transcript abundances of the bZIP transcription factors (TF) *ABA INSENSITIVE 5* (*BvABI5*) and *ABA RESPONSIVE ELEMENTS‐BINDING FACTOR 2* (*BvABF2*) decreased at 10°C and increased at 5°C, respectively, and this includes the secondary dormancy induction and maintenance period (Figure 4c). A distinct, but also temperature‐specific expression pattern was observed for the B3 TF *BvABI3/BvVP1*. The phosphatidylethanolamine‐binding protein, *MOTHER OF FT AND TFL1* (*MFT*), is known to mediate ABI5‐ABI3 and ABA‐GA interactions as well as responses at low temperature (Finch‐Savage & Footitt, 2017; Graeber et al., 2012; Vaistij et al., 2018). *BvMFT* transcripts were expressed with the same temperature‐dependent pattern as *BvABI5* and *BvABF2* (Figure 4c). Consistent with a specific role of ABI5 and MFT upregulation at 5°C in the secondary dormancy of KWS310, these genes are downregulated at 5°C in KWS257 (Figure 4d). High temperature suppresses *A. thaliana* seed germination by activating ABA biosynthesis, repressing GA biosynthesis and by ABI3‐ and ABI5‐mediated upregulation of the CCCH‐type zinc finger gene *SOMNUS* (*SOM*) (Lim et al., 2013). The gene expression patterns of *BvSOM* were in agreement with the roles of SOM in the cold‐induced secondary dormancy (Figure S8). *BvSOM* was upregulated at 5°C in KWS310 along with the accumulation ABA, and the upregulation of *BvABI3* and *BvABI5*.

**Figure 5:**
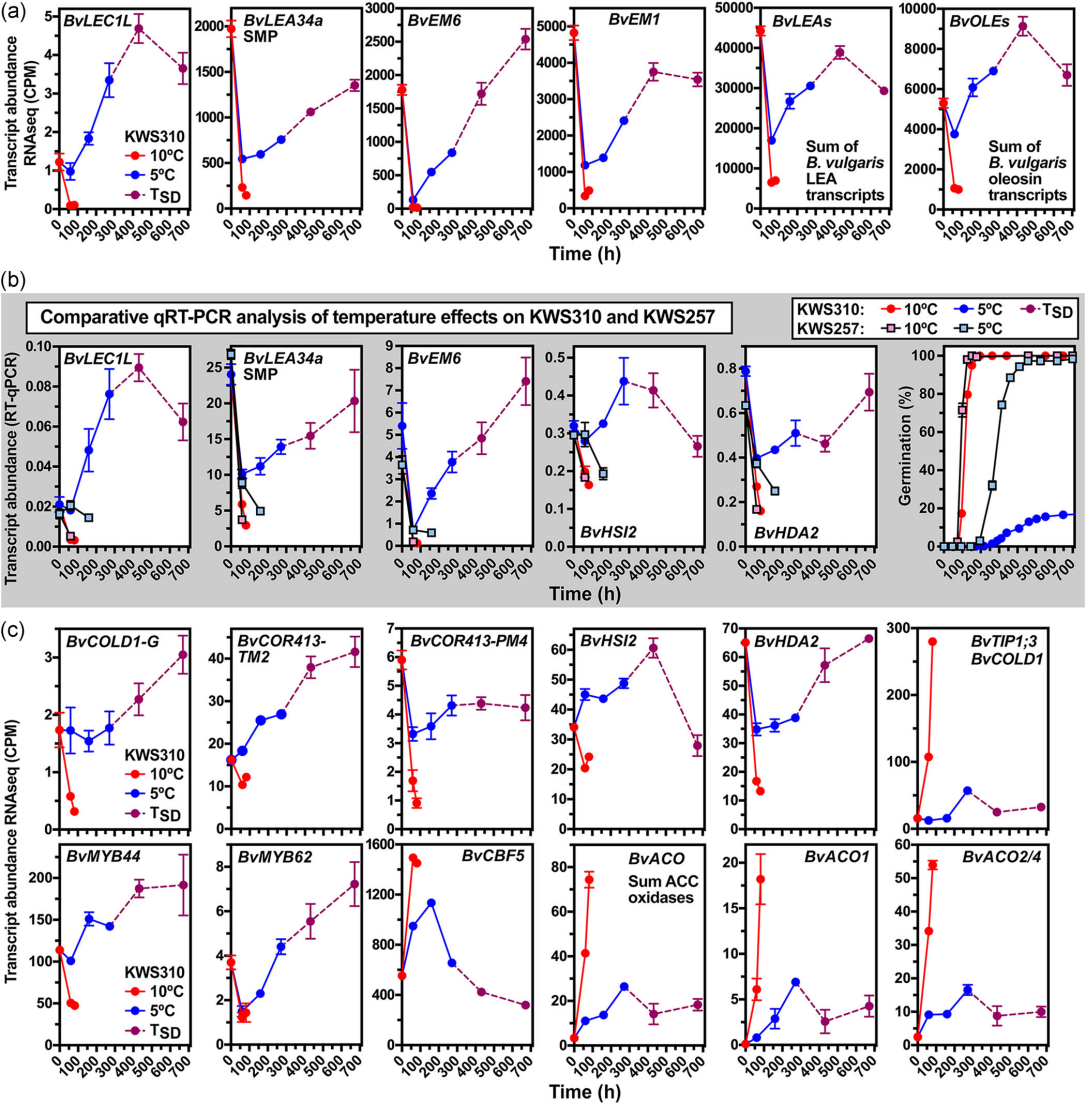
Gene expression patterns of major dormancy and maturation, histone modifiers and cold signalling components implicated in the cold‐induced secondary dormancy of sugar beet. Transcript abundances in sugar beet seeds incubated at the indicated temperature regimes: red (10°C), blue (5°C), purple (*T*_SD_), for details of the transcriptome analysis see Figure 4. For late embryogenesis abundant (*BvLEA*) and oleosin (*BvOLE*) genes, the sums of transcripts are presented. See Figures S8, S9 and S10 for additional genes, individual LEA genes and master regulator interactions, and Supporting Table S1 for the *Beta vulgaris* gene names and IDs. (a–c) Transcript abundances from the KWS310 transcriptome analysis. (b) RT‐qPCR analysis of transcript abundances of the contrasting KWS310 and KWS257 seedlots. See main text for gene abbreviations. Mean values ±SEM are presented. RT‐qPCR, reverse‐transcription quantitative polymerase chain reaction [Color figure can be viewed at wileyonlinelibrary.com]

The ABA‐related TFs may also have roles in the observed repression of GA biosynthesis in secondary dormant seeds (Figure S8). Both the upregulation of *GA 3‐OXIDASE* transcripts and the accumulation of bioactive GA_1_ and GA_4_ were repressed at 5°C compared to 10°C. As observed GA contents were very low, the induction of secondary dormancy was not a consequence of decreasing GA content. Ethylene is produced from its precursor 1‐aminocyclopropane‐1‐carboxylic acid (ACC). In sugar beet, ethylene produced by ACC oxidase (ACO) acts as an ABA antagonist to promote germination and early root growth (Abts et al., 2014; Hermann et al., 2007). In agreement with this, the expression of ACO genes was strongly upregulated during imbibition at 10°C (Figure 5c). In the low‐temperature regime (5°C), this upregulation was reduced and cold‐induced secondary dormancy induction and maintenance was associated with downregulation of *BvACO1* and *BvACO2/4* transcript abundances (Figure 5c). Taken together, ABA seems to play a key role in the hormonal regulation of cold‐induced secondary dormancy. Different cold signalling pathways may be involved in the secondary dormancy induction by chilling (Figures 5 and S8) as referred to in the Discussion section.

### 3.5 Expression of seed dormancy and maturation genes, and epigenetic regulation during sugar beet secondary dormancy

The ABA‐related bZIP TFs ABF2 and ABI5 bind to *cis*‐acting elements in promotor regions of downstream genes (Figure S9) to control maturation and dormancy programmes (Leprince et al., 2017). This includes genes for oleosins (OLE), late embryogenesis abundant (LEA), ‘early methionine‐labelled’ (EM), dehydrin (DHR) and seed maturation (SMP) proteins; named and assigned to distinct groups (LEA_1 to LEA_5, DHR, SMP) (Hundertmark & Hincha, 2008). In agreement with the germination programme, the transcript abundances of almost all oleosin and LEA genes decreased rapidly at 10°C in KWS310 (Figures 5 and S10). Contrastingly, they increased during imbibition at 5°C, upon secondary dormancy induction, and often also during secondary dormancy maintenance. The expression pattern of the master regulator of seed maturation *LEAFY COTYLEDON1‐LIKE* (*LEC1L*), a member of the highly conserved LEC1/LEC1L subfamily of NF‐YB TFs (Gnesutta et al., 2017; Jo et al., 2020; Leprince et al., 2017; Yamamoto et al., 2009), was consistent with the observed regulation of these maturation programme genes and suggests a key role in the processes. In KWS310 at 10°C the *BvLEC1* transcript abundance decreased rapidly, whereas at sup‐optimal conditions (5°C) and upon induction and maintenance of secondary dormancy *BvLEC1* transcripts accumulated (Figure 5). These patterns were verified for KWS310 by RT‐qPCR, which further demonstrated that in the contrasting line KWS257, the *BvLEC1*, *BvLEA34a* and *BvEM6* transcript abundances also decreased rapidly at 5°C (Figure 5b). The key dormancy gene *DELAY OF GERMINATION 1* (*DOG1*) (Bentsink et al., 2006; Bryant et al., 2019; Dekkers et al., 2016) was downregulated in sugar beet fruits upon imbibition and upregulated during dormancy maintenance (Figure S8). LEC1, DOG1, the ABA‐related TF described above and members of the ABA HYPERSENSITIVE GERMINATION1 (AHG1) subfamily of PP2Cs (AHG1/3, HAI1/2/3) are known to interact as regulators of maturation and dormancy programmes (Bryant et al., 2019; Dekkers et al., 2016; Leprince et al., 2017; Nee et al., 2017; Nishimura et al., 2018), which seems also evident during secondary dormancy (Figures 5, S8 and S9). Further, we discovered that major genes known to be involved in epigenetic control are regulated differentially by the temperature regimes and in association with secondary dormancy.

### 3.6 Imbibition temperature regulates cell‐wall remodelling gene expression required for embryo expansion and endosperm weakening

**Figure 6:**
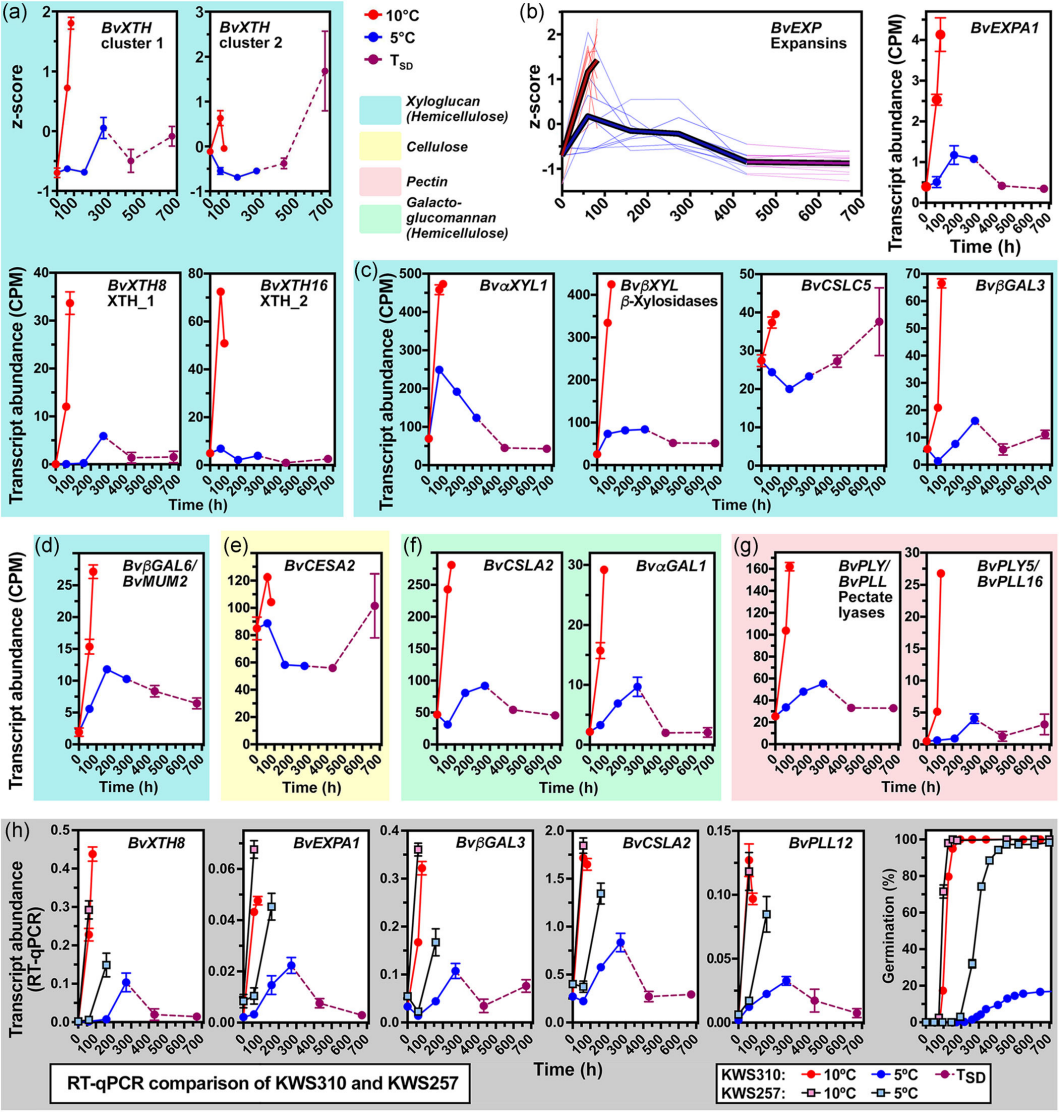
Gene expression patterns of cell wall remodelling proteins implicated in the cold‐induced secondary dormancy. Transcript abundances in sugar beet seeds incubated at the indicated temperature regimes: red (10°C), blue (5°C), and purple (*T*_SD_); for details of the transcriptome analysis, see Figure 4. For xyloglucan endotransglucosylase/hydrolases (*BvXTH*) and expansins (*BvEXP*), sums of transcripts are presented as z‐scores. See Figure S11 for additional genes, individual XTH genes and lists for the two XTH clusters, and Table S1 for the *Beta vulgaris* gene names and IDs. (a–g) Transcript abundances from the KWS310 transcriptome analysis. (h) RT‐qPCR analysis of transcript abundances of the contrasting KWS310 and KWS257 seedlots. See main text for gene abbreviations. Mean values ± SEM are presented. RT‐qPCR, reverse‐transcription quantitative polymerase chain reaction [Color figure can be viewed at wileyonlinelibrary.com]

The completion of germination requires biochemical cell wall loosening in embryo and endosperm cells by aROS/RBOH (Figure 2), expansins and cell wall remodelling proteins (Müller et al., 2009; Nonogaki, 2019; Scheler et al., 2015; Steinbrecher & Leubner‐Metzger, 2017). Figure 6 shows that the cold‐induced secondary dormancy is accompanied by massive changes in cell‐wall remodelling protein gene expression with all major cell wall polysaccharides (hemicelluloses, cellulose and pectin) as targets. Expansins were upregulated at 10°C (Figures 6b and S11). The rate of this upregulation is inhibited by the colder temperature (5°C) and subsequently, expansin gene expression was downregulated during initiation and maintenance of secondary dormancy in KWS310. This pattern in KWS310 was confirmed by RT‐qPCR, which also demonstrated that in the contrasting line KWS257 *BvEXPA1* is also upregulated at 5°C in association with its germination (Figure 6h).

Xyloglucan is the major hemicellulose in the primary cell walls of eudicots including sugar beet (Rose et al., 2002; Zykwinska et al., 2007). To achieve cell wall loosening, its chains are cleaved and reconnected by xyloglucan endotransglucosylase/hydrolases (XTHs). In imbibed sugar beet fruits, two clusters of XTH genes were identified based on co‐expression patterns (Figure 6a); gene names correspond to the closest *A. thaliana* homolog using the established nomenclature and groups of Rose et al. (2002). Cluster 1 XTH genes were rapidly upregulated upon imbibition at 10°C and expression levels increase until *T*_1%_, the onset of germination completion (Figure 6a). At the colder temperature, expression levels did not change until the 160 h timepoint at 5°C, but slightly increased with a small transient peak at *T*_1%_ (5°C; the 272 h timepoint), and subsequently remained lower during secondary dormancy induction and maintenance (Figures 6a and S11). At 10°C, cluster 2 XTH gene expression displayed upregulation upon imbibition but decreased again at *T*_1%_ (10°C). At 5°C, the expression of cluster 2 XTHs decreased and only later increased during secondary dormancy. Expression patterns of other xyloglucan, cellulose and galactoglucomannan remodelling enzymes are presented with detailed information in Figures 6 and S11. Their downregulation at 5°C in association with the secondary dormancy in KWS310 was verified by RT‐qPCR, which also demonstrated that in KWS257 these genes are upregulated at 5°C (Figure 6h).

Taken together these expression patterns demonstrate that distinct hemicellulose and cellulose remodelling is associated with germination and secondary dormancy of sugar beet. This is also true for the pectin component with pectate lyases (PLY/PLL) being important for cell wall loosening, cell separation and tissue weakening processes (Scheler et al., 2015; Sun & van Nocker, 2010; Uluisik & Seymour, 2020). Sun & van Nocker (2010) showed that in *A. thaliana* seeds the *PLL16* gene is expressed in the micropylar endosperm, which must weaken before the completion of germination by radicle protrusion (Steinbrecher & Leubner‐Metzger, 2017). While, for example, *BvPLL12* is downregulated in KWS310 at 5°C in association with the secondary dormancy, it is upregulated in KWS257 at 5°C in association with the germination programme (Figure 6h). Interestingly, and in agreement with this, transcripts of the *BvPLL16* homolog and three other *BvPLL* genes accumulated in sugar beet KWS310 seeds imbibed at 10°C, while at 5°C a much reduced initial and transient expression peak (similar to XTH cluster 1) was followed by downregulated upon secondary dormancy induction and maintenance (Figures 6g and S11). These findings suggest that the endosperm surrounding the radicle of sugar beet seeds (Hermann et al., 2007) may act as a temperature sensor.

## 4 DISCUSSION

### 4.1 Secondary dormancy induction by cold temperatures (chilling)

We report here that prolonged incubation of KWS310 sugar beet fruits at low temperature (5°C) induced secondary nondeep physiological dormancy in these apparently nondormant crops. Imbibition at low temperatures (cold‐stratification or prechilling, incubating imbibed seeds at 2–5°C for the purpose of overcoming blocks of dormancy) is well known for releasing the nondeep physiological dormancy of many species including *A. thaliana* (Auge et al., 2015; Baskin & Baskin, 2014; Walck et al., 2011; Yamauchi et al., 2004). In contrast to this, prolonged imbibition at hot temperatures causing thermoinhibition may induce thermodormancy in seeds of many species, including *A. thaliana* and lettuce (Argyris et al., 2008; Auge et al., 2015; Baskin & Baskin, 2014). Prolonged imbibition of *A. thaliana* seeds in the dark at 4°C did not induce secondary dormancy in the ecotype *Col‐0* which only has a shallow physiological dormancy (Auge et al., 2015), but did in the ecotype *Cvi* which has deeper physiological dormancy (Cadman et al., 2006). Beyond this, very few publications report examples for species in which secondary dormancy is induced by either low winter temperatures or imbibition at low temperature (4–5°C) in the laboratory (Banovetz & Scheiner, 1994; Baskin & Baskin, 1978; Hawkins et al., 2017). All these are wild species with a certain degree of dormancy, none of them are crop species and none of them produce primary nondormant seeds.

Seasonal dormancy cycling of *A. thaliana* in the soil seed bank requires passing through states of conditional dormancy (Baskin & Baskin, 1983; Cadman et al., 2006; Finch‐Savage & Footitt, 2017; Footitt et al., 2011; Footitt et al., 2020). To interpret changing seed responses to the environment requires a common general understanding of the seed dormancy continuum. Seed dormancy is a dynamic state in which the depth of dormancy of a single seed can have any value between all (maximum dormancy) and nothing (nondormancy). The concept of the dormancy continuum (conditional dormancy) illustrates this dynamic relationship between the depth of dormancy as a response to slow and fast signals in variable field environments (Baskin & Baskin, 2014; Finch‐Savage & Footitt, 2017; Soltani et al., 2019). It is agreed by many that dormancy exists as a continuum with a number of layers (blocks to germination completion) and that dormancy release involves their successive removal by appropriate environmental factors (e.g., temperature, moisture and light) and removal of the final layers permitting completion of germination (Finch‐Savage & Leubner‐Metzger, 2006). Each of these factors, therefore, removes successive blocks to germination, but this process usually needs to be carried out in a set order for it to work. Slow seasonal change is related to factors that are integrated over time to alter the depth of dormancy and the sensitivity to other factors. For *A. thaliana*, it has been shown that the depth of dormancy and key gene expression patterns are correlated with seasonal changes in soil temperature (Finch‐Savage & Footitt, 2017). Together with work into the volunteer crop weed oilseed rape (*B. napus*), reviewed by Soltani et al. (2019), our analysis of the physiological, hormonal, biomechanical and molecular mechanisms of the cold‐induced secondary dormancy of KWS310 fruits (*B. vulgaris*) supports the relevance of the concept of the dormancy continuum for crop species.

The contrasting sugar beet seedlots KWS310 (secondary dormancy at 5°C) and KWS257 (germination at 5°C) were produced under the same conditions (Figure S1), which suggests that genetic differences rather than the production environment per se are the primary reason of the observed difference. The primary objective for any breeding and seed‐producing company is the delivery of high‐quality seed, providing seed that germinates fast, uniformly and up to 100% under a broad range of sometimes adverse environmental conditions (Kockelmann et al., 2011). Field experiments of spring‐sown sugar beet indeed demonstrated that very early sowing (February) reduced the maximal field emergence depending on the year and cultivar (Hoffmann & Kluge‐Severin, 2011; Lamichhane et al., 2019). Chilling and freezing are among the weather‐related risks of early sowing with spring frosts increasing in Europe due to climate change (Gobin, 2018; Lamichhane et al., 2019; Zohner et al., 2020). The secondary dormancy in seedlot KWS310 was observed under laboratory conditions and a constant temperature regime of 5°C. Under field conditions, the soil temperature is fluctuating (Figure S1) and it remains unclear under which conditions a similar effect would be induced. The induction of secondary dormancy in response to chilling temperature conditions is an unwanted response as it could negatively affect yield generation if the percentage of emerged seedlings would be significantly reduced. To minimize this risk, appropriate varieties are used for production, and if required seed priming is used which alleviates dormancy (Figure 1) and increases emergence at low temperatures (Kockelmann et al., 2011). As with the volunteer crop weed oilseed rape (Soltani et al., 2019), weed beet can also become a serious problem in sugar beet fields. Its evolution in the soil seed bank has been well described including the role of dormancy (Sester et al., 2006, 2007, 2008), but the underpinning mechanisms have not been studied.

### 4.2 ABA‐related master dormancy and maturation regulators and the hormonal regulation of the cold‐induced secondary dormancy

Our working model (Figure S9) compiles the ABA‐associated master dormancy and maturation regulators, and epigenetic regulators. The observed distinct expression patterns support major roles in the responses to low temperature (5°C) and the induction and maintenance of secondary dormancy in KWS310, while KWS257 exhibits the germination programme also at 5°C. Cold‐induced upregulation of ABA biosynthesis (*BvZEP, BvNCED*) and downregulation of ABA degradation genes (*BvCYP707A*) leading to ABA accumulation in KWS310 (Figure 4) is well known in other species as the thermoinhibition response to seed imbibition at hot temperatures (Argyris et al., 2008; Lim et al., 2013; Toh et al., 2012). The ABA accumulation and expression of ABA metabolism genes are maintained during the cold‐induced secondary dormancy. This pattern is associated with the upregulation of gene expression for ABA‐related master dormancy and maturation regulators by the imbibition at 5°C (*BvABI3, BvABF2, BvABI5, BvMFT, BvSOM* and *BvLEC1*) known for their important roles in the seed dormancy and maturation programmes and during dormancy cycling of *A. thaliana* (Finch‐Savage & Footitt, 2017; Footitt et al., 2011; Graeber et al., 2012; Leprince et al., 2017; Lim et al., 2013; Nonogaki, 2017; North et al., 2010; Vaistij et al., 2018; Wilhelmsson et al., 2019; Yan et al., 2014). *BvABF2, BvABI5, BvMFT*, and *BvLEC1* transcript accumulation continues throughout the induction and maintenance (KWS310 in Figures 4 and 5) further supporting the importance of these ABA‐related master dormancy and maturation regulators in cold‐induced secondary dormancy. In contrast to this, germination programme expression patterns were evident for KWS257 also at 5°C.

LEC1/LEC1L and DOG1 were discovered as master regulators for seed maturation and dormancy, respectively (Bentsink et al., 2006; Bryant et al., 2019; Dekkers et al., 2016; Gnesutta et al., 2017; Leprince et al., 2017; Yamamoto et al., 2009). Their interaction with the ABA‐related master regulators ABI3, ABI5, AGH1/3 and ABF2 has been elucidated. During seed drying and maturation on the mother plant, they control the accumulation of oleosins and various types of LEA proteins (Hundertmark & Hincha, 2008; Leprince et al., 2017; Wilhelmsson et al., 2019). Recent work in *A. thaliana* demonstrates that LEC1 and temperature control *DOG1* expression (Dekkers et al., 2016), and that DOG1 also affects LEA protein accumulation (Bryant et al., 2019) during primary dormancy induction. We discovered here that during the cold‐induced secondary dormancy transcripts of numerous oleosin and LEA proteins of all groups accumulate (KWS310 in Figures 5, S9 and S10). LEA proteins may act by replacing water in dehydrating tissues (Hundertmark & Hincha, 2008; Leprince et al., 2017) and to our knowledge have not been described in seeds in the context of secondary dormancy. Their induction by chilling is however known from the onset of tree bud dormancy (Takemura et al., 2015). LEA proteins may also be involved in seed longevity (Leprince et al., 2017). We conclude for the cold‐induced secondary dormancy that although we did not use osmotica or seed drying, the maturation programme with oleosin and LEA protein accumulation was switched on in KWS310 as an integral process. In contrast to KWS310, the germination programme with downregulation of *BvABI5*, *BvMFT*, *BvLEC1L* and downstream *LEA* genes was evident in KWS257 also at 5°C. Epigenetic regulation and cold signalling of sugar beet secondary dormancy

4.3

Epigenetic regulation of seed dormancy and responses to cold temperature involve HISTONE MONO‐UBIQUITINATION 1 (HUB1) and HUB2 to alter DOG1 expression during seed dormancy cycling (Footitt et al., 2015; Graeber et al., 2012; Nonogaki, 2017) and regulate ABA biosynthesis via epigenetic regulation of *NCED* gene expression (Figure S9). Consistent with roles for histone modifications in secondary dormancy, *BvHUB1* and *BvHUB2* were downregulated at 10°C and induced during early imbibition in the cold temperature (Figure S8). At *T*_1%_ in 5°C, the expression levels of *BvHUB1/2* have decreased but then plateaued during induction and maintenance of secondary dormancy. The B3‐domain TFs HIGH‐LEVEL EXPRESSION OF SUGAR INDUCIBLE 2 (HSI2) and HSI2‐LIKE1 (HSL1) regulate seed dormancy, maturation and responses to cold temperature by controlling the expression of master maturation and dormancy regulator genes, including *LEC1*, *ABI3* and *DOG1* (N. C. Chen et al., 2020; Chhun et al., 2016; Nonogaki, 2017). This also involves histone modifications by HSI2 and HSL1 interacting in complexes with HISTONE DEACETYLASE2 (HDA2), HDA6, HDA9 or HDA19. The expression patterns of *BvHSI2*, *BvHSL1*, *BvHDA2*, *BvHDA9* and *BvHDA19* are consistent with roles for these TFs and histone modifications and distinct for KWS310 at the two temperature regimes (Figures 5 and S8). For *BvHSI2* and *BvHDA2* we also found contrasting expression patterns between KWS310 (secondary dormancy at 5°C) and KWS257 (germination at 5°C), which further supports the importance of histone modifications in the distinct regulation of the master maturation regulator expression in the two seedlots (Figures 5 and S9). During cold signalling, the B3 domain TF LEC2 competes with the repressive B3‐containing epigenome reader and Polycomb partner HSI2 for the *cis*‐regulatory cold memory element (CME) of the *FLOWERING LOCUS C* (*FLC*) gene to disrupt Polycomb signalling (Chung et al., 2021; Lepiniec et al., 2018; Tao et al., 2019). Further to this, the epigenetic regulator complexes involved in cold signalling also involve HAD6 (Chhun et al., 2016; Chung et al., 2021; Lepiniec et al., 2018). Chromatin‐based epigenetic mechanisms regulating the histone methylation and acetylation state of maturation regulators are therefore key to plant abiotic stress responses. The fact that *BvHSI2* and *BvHDA2* are differentially expressed at low temperature (5°C) between KWS310 and KWS257 (Figures 5 and S9) point to a key role of histone modifications in the chilling‐induced secondary dormancy and thereby to an important area of future research into its epigenetic regulation.

Cold signalling in the endosperm determines the depth of primary seed dormancy in *A. thaliana* and is associated with ABI3‐mediated increase in endospermic ABA contents (Chahtane et al., 2017; MacGregor et al., 2019; Yan et al., 2014). MYB‐type TF are also involved in this cold signalling response, as well as downstream cold‐responsive (COR) genes of the classical C‐repeat‐Binding Factor (CBF) pathway (Chang et al., 2020; Y. K. Liu, Dang, et al., 2019; Park et al., 2018). In agreement with the role of these cold signalling pathways in secondary dormancy, *BvMYB62*, *BvMYB44*, *BvCBF5* and two *BvCOR413‐TM2* are expressed in a cold‐induced manner in sugar beet fruits (Figure 5). The *OsCOLD1* gene confers chilling tolerance in rice and encodes a GPCR‐type G protein (Ma et al., 2015). Transcript expression of *BvCOLD1‐G*, the sugar beet homolog of this chilling tolerance gene, was upregulated in the 5°C temperature regime and during secondary dormancy and downregulated at 10°C (Figure 5). Key aquaporin genes of the tonoplast intrinsic protein (TIP) group are known to be involved in water uptake during seed germination (Footitt et al., 2019). BvTIP1;3/BvCOLD1 was demonstrated to be a sugar beet aquaporin that confers cold tolerance (Porcel et al., 2018). Figure 5 shows that the transcript abundances of BvTIP1;3/BvCOLD1 are rapidly upregulated in germinating sugar beet fruits imbibed at 10°C, whereas at 5°C the upregulation was at a reduced rate until *T*_1%_ and a subsequent downregulation was observed along with the induction of secondary dormancy. Distinct expression patterns in the two temperature regimes were also observed for other seed TIPs (Figure S8). These findings suggest that different cold signalling pathways may be involved in the secondary dormancy induction by chilling. To elucidate their interaction with components of the identified ABA signalling, dormancy and maturation modules during the chilling‐induced secondary dormancy (Figure S9) is another important area for future research. Cold signalling via the CBF pathway also involves epigenetic regulation including histone modifications (Chang et al., 2020; Y. K. Liu et al., 2019b; Park et al., 2018), and further supports that this is an important area of future research.

### 4.4 The cold‐induced secondary dormancy involves inhibition of cell‐wall remodelling gene expression required for endosperm weakening and embryo growth

The contrasting responses and mechanisms to low temperature (5°C) of KWS310 (secondary dormancy programme) and KWS257 (germination programme) were also clearly evident from the distinct expression patterns of the downstream genes, which control endosperm weakening and embryo growth (Figures 2, 6, S9). In KWS310 fruits, many of the cell‐wall remodelling genes exhibited an expression pattern similar to cluster 1‐type *BvXTHs* (Figure 6a): At 10°C upregulation and at 5°C a severely reduced rate of upregulation, a small transient peak around *T*_1%_ and subsequent downregulation during secondary dormancy induction and maintenance. Our interpretation for the small transient peak at *T*_1%_ (5°C) is that this is caused by the small fraction of KWS310 fruits (~18%, Figure 1a) that is able to germinate at 5°C. This is further supported by their upregulation at 5°C in KWS257 fruits, which is in association with its germination programme (Figure 6h). Consequently, this suggests that these are genes upregulated in the germination programme and downregulated in the secondary dormancy programme. This expression pattern is evident for many expansins, XTHs, xylosidases, galactosidases, RBOHs and pectate lyases (Figures 2, 6, S8, S9). Taken together this suggests that secondary dormancy in sugar beet is controlled by massive changes in cell‐wall remodelling protein gene expression with all major cell wall polysaccharides (hemicelluloses, cellulose, pectin) as targets. This is known to affect the biomechanics of embryo elongation and endosperm weakening of many other species (Graeber et al., 2014; Müller et al., 2009; Nonogaki, 2019; Rodriguez‐Gacio Mdel et al., 2012; Steinbrecher & Leubner‐Metzger, 2017).

From a mechanistic point of view, dormancy and germination are controlled by two opposing forces, the embryo growth potential and the tissue restraint imposed by the seed‐covering layers (Nonogaki, 2006; Steinbrecher & Leubner‐Metzger, 2017; Yan et al., 2014). We demonstrate here that at their respective *T*_1%_ (82 h for 10°C; 272 h for 5°C) the sugar beet embryo growth potential (EGP) was already reduced by the chilling and subsequent to the establishment of secondary dormancy this reduction is further intensified. The embryos isolated from the cold‐induced secondary dormant seeds do not fully lose their capacity to grow at 0 MPa (pure water), but they do grow at a far slower rate and even a slight reduction of the water potential (−0.1 MPa) results in a complete cessation of growth. Examples for cell‐wall remodelling genes proposed to be involved in altering seed biomechanics include: α‐xylosidase in seeds of a thermoinhibition‐resistant *A. thaliana* mutant altered in dormancy and cell wall mechanics and α‐xylosidase enzyme activity in the endosperm (Sechet et al., 2016; Shigeyama et al., 2016); XTHs, expansins and other cell‐wall remodelling proteins expressed in the micropylar endosperm of seeds of tomato, coffee, lettuce and other species (F. Chen & Bradford, 2000; F. Chen et al., 2002; da Silva et al., 2004; Steinbrecher & Leubner‐Metzger, 2017); and pectate lyases expressed in the micropylar endosperm of *A. thaliana* seeds (Sun & van Nocker, 2010; Uluisik & Seymour, 2020). Their altered expression patterns in sugar beet in response to low temperature, accumulating ABA and during seed dormancy provide a link to the altered biomechanics of embryo elongation and endosperm weakening. In mature sugar beet fruits, the radicle is surrounded by an endosperm (Hermann et al., 2007), and it has been proposed that the endosperm tissue is a major mediator of communication between the embryo and the environment (Yan et al., 2014). ABA accumulation, altered gene expression and inhibition of endosperm weakening suggest that the endosperm may act as a temperature sensor involved in cold‐induced secondary dormancy. Earlier sowing of seed due to climatic change makes crop establishment potentially vulnerable to early season frost (Finch‐Savage & Bassel, 2016; Moore & Lobell, 2015). Mitigating this risk by seed priming technologies (Figure 1) and breeding chilling tolerant cultivars not prone to cold‐induced secondary dormancy is therefore important for climate‐smart agriculture and food security.

## CONFLICT OF INTERESTS

The authors declare that there are no conflict of interests.

## Supporting information

Supplementary information.

Supplementary information.

Supplementary information.

Supplementary information.

## Data Availability

RNA‐seq data have been deposited in the National Center for Biotechnology Information Sequence Read Archive (NCBI SRA) with BioProject number PRJNA675466 (https://www.ncbi.nlm.nih.gov/bioproject/?term=PRJNA675466). All other study data presented or analysed in this article and Supporting Data is available online through figshare https://doi.org/10.17637/rh.13207973.
