# Supplementary material for: Cold‐induced secondary dormancy and its regulatory mechanisms in Beta vulgaris
Source: Plant Cell Environ. 2022 Jan 28;45(4):1315–32. doi: 10.1111/pce.14264 (PMC9305896; doi:10.1111/pce.14264)
Supplement: Supplementary file 2 — Supplementary information. [file PCE-45-1315-s001.docx]

Cold-induced secondary dormancy and its regulatory mechanisms in *Beta vulgaris*

James E. Hourston^1,#^, Tina Steinbrecher^1,#^, Jake O. Chandler^1^, Marta Pérez^1^, Katrin Dietrich^2^, Veronika Turečková^3^, Danuše Tarkowská^3^, Miroslav Strnad^3^, Fridtjof Weltmeier^2^, Juliane Meinhard^2^, Uwe Fischer^2^, Karin Fiedler-Wiechers^2^, Michael Ignatz^1^, Gerhard Leubner-Metzger^1,3^

^1^Department of Biological Sciences, Royal Holloway University of London, Egham, TW20 0EX, United Kingdom; Web: 'The Seed Biology Place' - www.seedbiology.eu

^2^KWS SAAT SE & Co. KGaA, Grimsehlstr. 31, D-37555 Einbeck, Germany

^3^Laboratory of Growth Regulators, Palacký University and Institute of Experimental Botany, Czech Academy of Sciences, CZ-78371 Olomouc, Czech Republic

^#^These authors contributed equally to this work.

Correspondence (shared corresponding authors)

Gerhard Leubner-Metzger and Michael Ignatz, Department of Biological Sciences, Royal Holloway University of London, Egham, TW20 0EX, United Kingdom.

Web: 'The Seed Biology Place' - www.seedbiology.eu

Email: gerhard.leubner@rhul.ac.uk (G.L.-M.) and ignatz.michael@gmail.com (M.I.)

**Plant, Cell & Environment:** Original Article

**Running title:** Cold-induced secondary dormancy

SUPPORTING INFORMATION

This is an open access article under the terms of the Creative Commons Attribution CC-BY 4.0 License, which permits use, distribution and reproduction in any medium, provided the original work is properly cited.

© 2021 The Authors, *Plant, Cell & Environment* published by Wiley & Sons Ltd.

Supporting Methods

**Embryo growth potential (EGP) assays**

To determine embryo growth potential ungerminated fruits were incubated in white boxes at 5ºC or 10ºC. At the physiological time point whereupon both sets of fruits were at ~1% (T_1%_) of their potential germination the fruits were dissected, the pericarp, testa and perisperm removed and the embryo alone transferred to petri dishes with 2 filter papers and 4 ml dH_2_O or 10, 20, 30, 40 mM polyethylene glycol (PEG) 6000 (Carl Roth GmbH & Co. KG, Germany) and incubated at 10ºC in darkness. Ungerminated fruits, also incubated at 5 °C but until maximal germination (~18%) had occurred at ~750h provided a set of embryos that had acquired secondary dormancy. To measure the length of the radicle-hypocotyl axis, embryos were transferred to a black background during the incubation period at 10°C after 50 ± 2.5 h and photographed using a digital camera (Leica DFC480 with Leica IM1000 software) connected to a Leica MZ12_5_ (Leica Biosystems, Germany) stereo microscope with 8-fold magnification. Twenty-four-bit RGB images at a resolution of 2560 × 1920 pixel (pixel aspect ratio = 1) were saved in JPG format. Image J (Schneider et al., 2012) was used to quantify the embryo growth.

**Pre-emergence seedling growth assays**

To investigate pre-emergence seedling growth independently of variability in germination timing a suitable assay was developed (Figure S4a). Fruits were imbibed at 10ºC (standard germination assay conditions) until the radicle emergence stage. This stage with radicle emergence just completed was selected as "day 0" for the pre-emergence seedling growth assay. "Day 0" fruits were transferred to a vertical agar plate system used to monitor seedling growth over time at either 10ºC or 5ºC (Figure S4a). Seedling growth was analysed using Image J as described above.

**Histochemical staining for apoplastic Reactive Oxygen Species (aROS)**

The histochemical staining assay with *p*-nitrotetrazolium blue chloride (NBT) was used to visualise apoplastic superoxide (Oracz et al., 2012). Fifteen sugar beet embryos were isolated from ungerminated fruits. The incubation time at 10ºC was 150 h, incubation at 5ºC was until after maximum germination for 5ºC was reached at 460 h. The 5ºC embryos were additionally incubated for 6 days at 10ºC. Isolated embryos were equilibrated (for 20 mins) on ice in 10 mM potassium phosphate buffer (pH 6.6). Thereafter they were transferred into the staining solution. Embryos were incubated in 500 µl NBT solution (6 mM; Roth 4421.3; dissolved in 10 mM potassium phosphate buffer, pH 6.6) for 8 mins at room temperature. Embryos were then removed from the staining solution and washed for 1 min in phosphate buffer. Images were taken with a stereomicroscope (MZ12_5_ Leica Biosystems, Germany) with integrated camera (Leica DFC480 with Leica IM1000 software).

**Phytohormone quantification**

Fruits were sampled in the dry state, and then following imbibition at either 5ºC or 10ºC, at the times indicated in the times indicated (Figure 4; Supplemental Figure S4). The pericarps were removed prior to snap freezing of the true seeds in liquid nitrogen (n = 5 x 100 seeds), at which point they were ground, and lyophilized. Five replicates (each 20 mg) of this powder was used for the quantification of *cis*-(+)-abscisic acid (ABA), gibberellins (GA) and GA metabolites. **Abscisic acid:** Samples were extracted in 1 ml cold methanol/water/acetic acid (10/89/1, v/v/v) and 20 pmol of [^2^H_6_](+)ABA (Olchemim) was added to the samples as internal standard. After 1 h, the homogenates were centrifuged and the pellets were re-extracted for 30 min as described by Turečková et al. (Turečková et al., 2009). The combined supernatants were cleaned on an Oasis® HLB cartridges (60 mg, 3 ml, Waters, Milford, MA, USA), then evaporated to dryness in a Speed-Vac (UniEquip) and finally analyzed by UPLC-ESI(-)-MS/MS (Turečková et al., 2009). **Gibberellins:** The levels of 20 GA derivatives, physiologically active or non‐active were quantified as described by Urbanová et al (Urbanova et al., 2013).

**Transcriptome analysis**

For the transcriptome analyses true seeds (4 x 100) extracted from KWS310 fruits were sampled when dry, and following imbibition at the time points specified (Figure 3a). RNA was extracted using methods outlined in Graeber et al (2011) with the modification that 20 mg of true seed powder was used for each biological replicate and additional grinding was performed by 2 x 12 sec with screw top tubes and ceramic beads in a Precellys Evolution homogenizer (Bertin Technologies SAS, Montigny-le-Bretonneux, France). RNA quantity and purity were determined with the Nanodrop ND-1000 (Peqlab), and RNA integrity checked using Agilent 2100 Bioanalyzer with the RNA 6000 Nano chip and Plant RNA Nano protocol. Samples used had absorbance ratios of at least 2 (A_260/280 nm_) and 1.8 (A_260/230 nm_) and a RIN ≥ 8.4. Three biological replicates of RNA samples were used for downstream applications. For conducting RNA-seq, strand specific cDNA libraries were prepared following polyA selection and paired-end (2 x 100 bp) sequencing was performed with Illumina HiSeq 2500 chemistry v4 in high-output run mode (Eurofins Genomics GmBH, Ebersberg, Germany). Reads were mapped using STAR aligner (Dobin et al., 2013) against RefBeet-1.2 (Dohm et al., 2014). Mapped reads were counted with featureCounts (Liao et al., 2014) for BeetSet-2 gene models (Minoche et al., 2015). Differential gene expression was calculated in R using DESeq2 (version 1.18.1) (Love et al., 2014). Genes with very few read counts (sum counts across all samples < 30) were excluded from the analysis. Log_2_ fold change (Log2FC) and adjusted p-value (FDR adjusted p-value were calculated using Benjamini and Hochberg method (Benjamini & Hochberg, 1995). Genes with a log2FC > 1 and adjusted *p*-value of < 0.05 or genes with log2FC < -1 and adjusted *p*-value of < 0.05 were considered as differentially expressed genes (DEGs).

Supporting References

Benjamini, Y., & Hochberg, Y. (1995). Controlling the false discovery rate: a practical and powerful approach to multiple testing. *J R Stat Soc B, 57*, 289–300.

Bentsink, L., Jowett, J., Hanhart, C. J., & Koornneef, M. (2006). Cloning of *DOG1*, a quantitative trait locus controlling seed dormancy in Arabidopsis. *PNAS, 103*(45), 17042-17047.

Bradford, K. J., Downie, A. B., Gee, O. H., Alvarado, V., Yang, H., & Dahal, P. (2003). Abscisic acid and gibberellin differentially regulate expression of genes of the SNF1-related kinase complex in tomato seeds. *Plant Physiology, 132*(3), 1560-1576.

Bryant, F. M., Hughes, D., Hassani-Pak, K., & Eastmond, P. J. (2019). Basic LEUCINE ZIPPER TRANSCRIPTION FACTOR67 transactivates DELAY OF GERMINATION1 to establish primary seed dormancy in Arabidopsis. *Plant Cell, 31*(6), 1276-1288.

Chen, N. C., Wang, H., Abdelmageed, H., Veerappan, V., Tadege, M., & Allen, R. D. (2020). HSI2/VAL1 and HSL1/VAL2 function redundantly to repress DOG1 expression in Arabidopsis seeds and seedlings. *New Phytologist, 227*(3), 840–856.

Chhun, T., Chong, S. Y., Park, B. S., Wong, E. C. C., Yin, J. L., Kim, M., & Chua, N. H. (2016). HSI2 repressor recruits MED13 and HDA6 to down-regulate seed maturation gene expression directly during Arabidopsis early seedling growth. *Plant and Cell Physiology, 57*(8), 1689-1706.

Deihimfard, R., Rahimi-Moghaddam, S., & Chenu, K. (2019). Risk assessment of frost damage to sugar beet simulated under cold and semi-arid environments. *International Journal of Biometeorology, 63*(4), 511-521.

Dekkers, B. J., He, H., Hanson, J., Willems, L. A., Jamar, D. C., Cueff, G., . . . Bentsink, L. (2016). The Arabidopsis DELAY OF GERMINATION 1 gene affects ABSCISIC ACID INSENSITIVE 5 (ABI5) expression and genetically interacts with ABI3 during Arabidopsis seed development. *Plant J, 85*(4), 451-465.

Dobin, A., Davis, C. A., Schlesinger, F., Drenkow, J., Zaleski, C., Jha, S., . . . Gingeras, T. R. (2013). STAR: ultrafast universal RNA-seq aligner. *Bioinformatics, 29*(1), 15-21.

Dohm, J. C., Minoche, A. E., Holtgrawe, D., Capella-Gutierrez, S., Zakrzewski, F., Tafer, H., . . . Himmelbauer, H. (2014). The genome of the recently domesticated crop plant sugar beet (*Beta vulgaris*). *Nature, 505-509*(7484), 546.

Durr, C., & Boiffin, J. (1995). Sugar beet seedling growth from germination to first leaf stage. *Journal of Agricultural Science, 124*, 427-435.

Finch-Savage, W. E., & Footitt, S. (2017). Seed dormancy cycling and the regulation of dormancy mechanisms to time germination in variable field environments. *Journal of Experimental Botany, 68*(4), 843-856.

Finch-Savage, W. E., & Leubner-Metzger, G. (2006). Seed dormancy and the control of germination. *New Phytologist, 171*, 501-523.

Gnesutta, N., Saad, D., Chaves-Sanjuan, A., Mantovani, R., & Nardini, M. (2017). Crystal structure of the *Arabidopsis thaliana* L1L/NF-YC3 histone-fold dimer reveals specificities of the LEC1 family of NF-Y subunits in plants. *Molecular Plant, 10*(4), 645-648.

Graeber, K., Linkies, A., Steinbrecher, T., Mummenhoff, K., Tarkowská, D., Turečková, V., . . . Leubner-Metzger, G. (2014). *DELAY OF GERMINATION 1* mediates a conserved coat dormancy mechanism for the temperature- and gibberellin-dependent control of seed germination. *PNAS, 111*, E3571-E3580.

Graeber, K., Linkies, A., Wood, A. T., & Leubner-Metzger, G. (2011). A guideline to family-wide comparative state-of-the-art quantitative RT-PCR analysis exemplified with a Brassicaceae cross-species seed germination case study. *The Plant Cell, 23*, 2045-2063.

Graeber, K., Nakabayashi, K., Miatton, E., Leubner-Metzger, G., & Soppe, W. J. (2012). Molecular mechanisms of seed dormancy. *Plant, Cell & Environment, 35*, 1769-1786.

Gummerson, R. J. (1986). The effect of constant temperatures and osmotic potential on the germination of sugar beet. *Journal of Experimental Botany, 37*, 729–958.

Hilhorst, H. W. M., & Downie, B. (1995). Primary dormancy in tomato (*Lycopersicon esculentum* cv. Moneymaker): Studies with the *sitiens* mutant. *Journal of Experimental Botany, 47*(294), 89-97.

Hundertmark, M., & Hincha, D. K. (2008). LEA (late embryogenesis abundant) proteins and their encoding genes in *Arabidopsis thaliana*. *BMC Genomics, 9*, 118.

Jo, L., Pelletier, J. M., Hsu, S. W., Baden, R., Goldberg, R. B., & Harada, J. J. (2020). Combinatorial interactions of the LEC1 transcription factor specify diverse developmental programs during soybean seed development. *PNAS, 117*(2), 1223-1232.

Kockelmann, A., Tilcher, R., & Fischer, U. (2011). Seed production and processing. *Sugar TEch, 12*(3), 267-275.

Lamichhane, J. R., Constantin, J., Aubertot, J. N., & Durr, C. (2019). Will climate change affect sugar beet establishment of the 21st century? Insights from a simulation study using a crop emergence model. *Field Crops Research, 238*, 64-73.

Leprince, O., Pellizzaro, A., Berriri, S., & Buitink, J. (2017). Late seed maturation: drying without dying. *Journal of Experimental Botany, 68*(4), 827-841.

Liao, Y., Smyth, G. K., & Shi, W. (2014). FeatureCounts: an efficient general purpose program for assigning sequence reads to genomic features. *Bioinformatics, 30*(7), 923-930.

Linkies, A., & Leubner-Metzger, G. (2012). Beyond gibberellins and abscisic acid: how ethylene and jasmonates control seed germination. *Plant Cell Reports, 31*(2), 253-270.

Liu, Y. K., Dang, P. Y., Liu, L. X., & He, C. Z. (2019). Cold acclimation by the CBF-COR pathway in a changing climate: Lessons from *Arabidopsis thaliana*. *Plant Cell Reports, 38*(5), 511-519.

Love, M. I., Huber, W., & Anders, S. (2014). Moderated estimation of fold change and dispersion for RNA-seq data with DESeq2. *Genome Biology, 15*(12).

Ma, Y., Dai, X. Y., Xu, Y. Y., Luo, W., Zheng, X. M., Zeng, D. L., . . . Chong, K. (2015). COLD1 confers chilling tolerance in rice. *Cell, 160*(6), 1209-1221.

Minoche, A. E., Dohm, J. C., Schneider, J., Holtgrawe, D., Viehover, P., Montfort, M., . . . Himmelbauer, H. (2015). Exploiting single-molecule transcript sequencing for eukaryotic gene prediction. *Genome Biology, 16*, 184.

Müller, K., Linkies, A., Vreeburg, R. A. M., Fry, S. C., Krieger-Liszkay, A., & Leubner-Metzger, G. (2009). *In vivo* cell wall loosening by hydroxyl radicals during cress (*Lepidium sativum* L.) seed germination and elongation growth. *Plant Physiology, 150*, 1855-1865.

Nakashima, K., Fujita, Y., Kanamori, N., Katagiri, T., Umezawa, T., Kidokoro, S., . . . Yamaguchi-Shinozaki, K. (2009). Three Arabidopsis SnRK2 protein kinases, SRK2D/SnRK2.2, SRK2E/SnRK2.6/OST1 and SRK2I/SnRK2.3, involved in ABA signaling are essential for the control of seed development and dormancy. *Plant & Cell Physiology, 50*, 1345-1363.

Nee, G., Kramer, K., Nakabayashi, K., Yuan, B. J., Xiang, Y., Miatton, E., . . . Soppe, W. J. J. (2017). DELAY OF GERMINATION1 requires PP2C phosphatases of the ABA signalling pathway to control seed dormancy. *Nature Communications, 8*, ARTN 72.

Nishimura, N., Tsuchiya, W., Moresco, J. J., Hayashi, Y., Satoh, K., Kaiwa, N., . . . Yamazaki, T. (2018). Control of seed dormancy and germination by DOG1-AHG1 PP2C phosphatase complex via binding to heme. *Nature Communications, 9*, ARTN 2132.

Nonogaki, H. (2017). Seed biology updates - highlights and new discoveries in seed dormancy and germination research. *Front Plant Sci, 8*, 524.

Oracz, K., Voegele, A., Tarkowska, D., Jacquemoud, D., Tureckova, V., Urbanova, T., . . . Leubner-Metzger, G. (2012). Myrigalone A inhibits *Lepidium sativum* seed germination by interference with gibberellin metabolism and apoplastic superoxide production required for embryo extension growth and endosperm rupture. *Plant & Cell Physiology, 53*, 81-95.

Rodriguez-Gacio Mdel, C., Iglesias-Fernandez, R., Carbonero, P., & Matilla, A. J. (2012). Softening-up mannan-rich cell walls. *Journal of Experimental Botany, 63*(11), 3976-3988.

Rose, J. K., Braam, J., Fry, S. C., & Nishitani, K. (2002). The XTH family of enzymes involved in xyloglucan endotransglucosylation and endohydrolysis: current perspectives and a new unifying nomenclature. *Plant & Cell Physiology, 43*(12), 1421-1435.

Salimi, Z., & Boelt, B. (2019). From emergence to flowering: four beet (*Beta vulgaris* ssp.) cultivars' phenological response to seed priming. *Agronomy-Basel, 9*(12), ARTN 863.

Schneider, C. A., Rasband, W. S., & Eliceiri, K. W. (2012). NIH Image to ImageJ: 25 years of image analysis. *Nat Methods, 9*(7), 671-675.

Sechet, J., Frey, A., Effroy-Cuzzi, D., Berger, A., Perreau, F., Cueff, G., . . . Marion-Poll, A. (2016). Xyloglucan metabolism differentially impacts the cell wall characteristics of the endosperm and embryo during Arabidopsis seed germination. *Plant Physiology, 170*(3), 1367-1380.

Shigeyama, T., Watanabe, A., Tokuchi, K., Toh, S., Sakurai, N., Shibuya, N., & Kawakami, N. (2016). alpha-Xylosidase plays essential roles in xyloglucan remodelling, maintenance of cell wall integrity, and seed germination in *Arabidopsis thaliana*. *Journal of Experimental Botany, 67*(19), 5615-5629.

Turečková, V., Novák, O., & Strnad, M. (2009). Profiling ABA metabolites in *Nicotiana tabacum* L. leaves by ultra-performance liquid chromatography-electrospray tandem mass spectrometry. *Talanta, 80*, 390-399.

Urbanova, T., Tarkowska, D., Novak, O., Hedden, P., & Strnad, M. (2013). Analysis of gibberellins as free acids by ultra performance liquid chromatography-tandem mass spectrometry. *Talanta, 112*, 85-94.

Vaistij, F. E., Barros-Galvao, T., Cole, A. F., Gilday, A. D., He, Z. S., Li, Y., . . . Graham, I. A. (2018). MOTHER-OF-FT-AND-TFL1 represses seed germination under far-red light by modulating phytohormone responses in *Arabidopsis thaliana*. *PNAS, 115*(33), 8442-8447.

Wilhelmsson, P. K. I., Chandler, J. O., Fernandez-Pozo, N., Graeber, K., Ullrich, K. K., Arshad, W., . . . Rensing, S. A. (2019). Usability of reference-free transcriptome assemblies for detection of differential expression: a case study on *Aethionema arabicum* dimorphic seeds. *BMC Genomics, 20*, ARTN 95.

Yamamoto, A., Kagaya, Y., Toyoshima, R., Kagaya, M., Takeda, S., & Hattori, T. (2009). Arabidopsis NF-YB subunits LEC1 and LEC1-LIKE activate transcription by interacting with seed-specific ABRE-binding factors. *Plant Journal, 58*(5), 843-856.

Yan, D., Duermeyer, L., Leoveanu, C., & Nambara, E. (2014). The functions of the endosperm during seed germination. *Plant & Cell Physiology, 55*(9), 1521-1533.

**
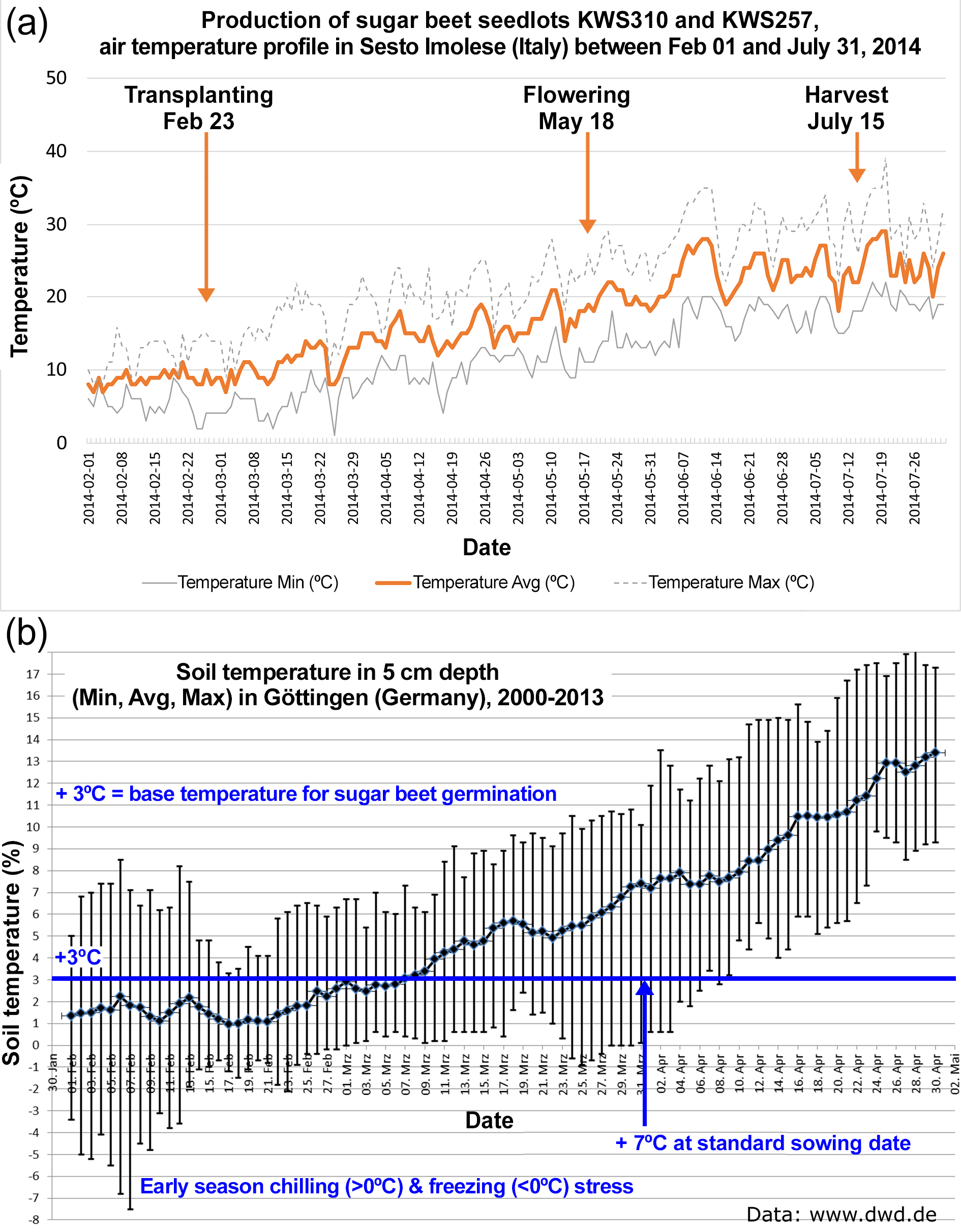
**

**Figure S1. Temperatures during sugar beet commercial seed production and sowing.** (a) Air temperature profile during the production of seedlots KWS310 and KWS257 as an example for the typical production environment for hybrid sugar beet seeds (Kockelmann et al., 2011). Both seedlots were produced according to commercial standards in the region of Sesto Imolese, Italy in the year 2014. Basic seed from both parental lines was sown in specific nursery fields in 2013 and grown to small beet plants (= stecklings) which were transplanted to the production field on February 23, 2014. Having received a vernalisation treatment, bolting was induced to enable flowering of the fertile seed parent as well as of the sterile pollen donor. Flowering started on May 18 and seeds were harvested in July 15. During flowering and seed development, daily mean temperatures were typically between 20 and 30ºC, while maximum temperatures frequently were often above 30ºC. Temperature data were provided by the Ingentis data management system. (b) Early season soil temperatures in a sugar beet growing region in Göttingen, Germany. The base temperature for sugar beet seed germination is ca. 3ºC (Durr & Boiffin, 1995; Gummerson, 1986; Lamichhane et al., 2019), and the standard sowing date end of March is characterized by ca. 7ºC as average soil temperature. Earlier sowing to benefit from an extended growing season due to climate change bears the higher risk of early season chilling and frost stress during the vulnerable stages of crop establishment (Deihimfard et al., 2019; Lamichhane et al., 2019). Past and future sowing date adaptation is an important change to increase yield potential: the average sowing date for sugar beet in the 1980s was mid-April while it became mid-March nowadays.

**
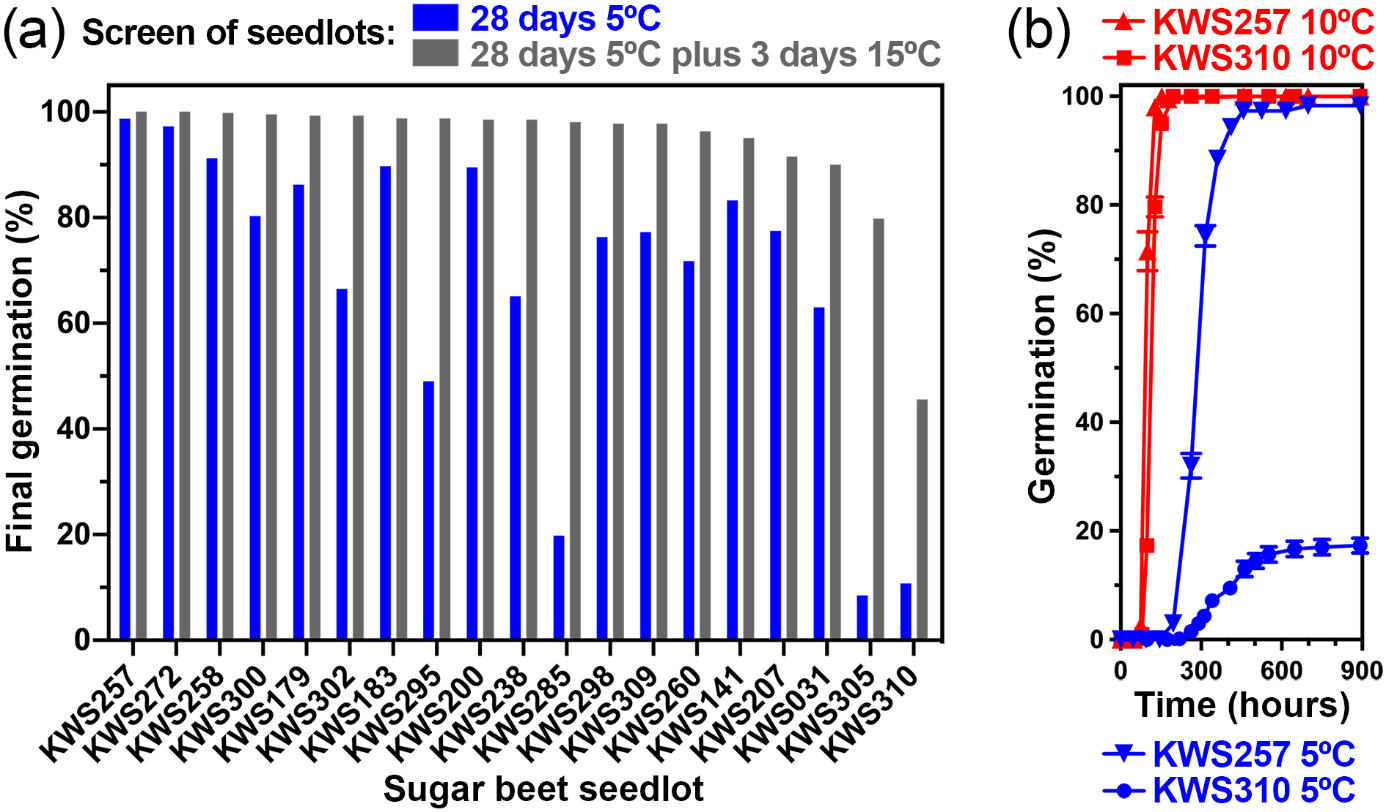
**

**Figure S2.** **Screening germination responses to chilling of sugar beet seedlots and identification of cold-induced secondary dormancy in seedlot KWS310.** (a) Final germination percentages in a screening of 19 sugar beet fruit lots incubated for 28 days at 5ºC (blue columns) compared to an additional subsequent incubation for 3 days at 15ºC (grey columns). Note that only seedlot KWS310 revealed a substantial induction of secondary dormancy upon chilling. (b) Verification of the germination responses for KWS310 demonstrated that ca. 80% of fruits did not germinate even after 37 days incubation at 5ºC. In contrast to this KWS257 fully germinated within less than 10 days at 5ºC; mean values ± SEM are presented.

**
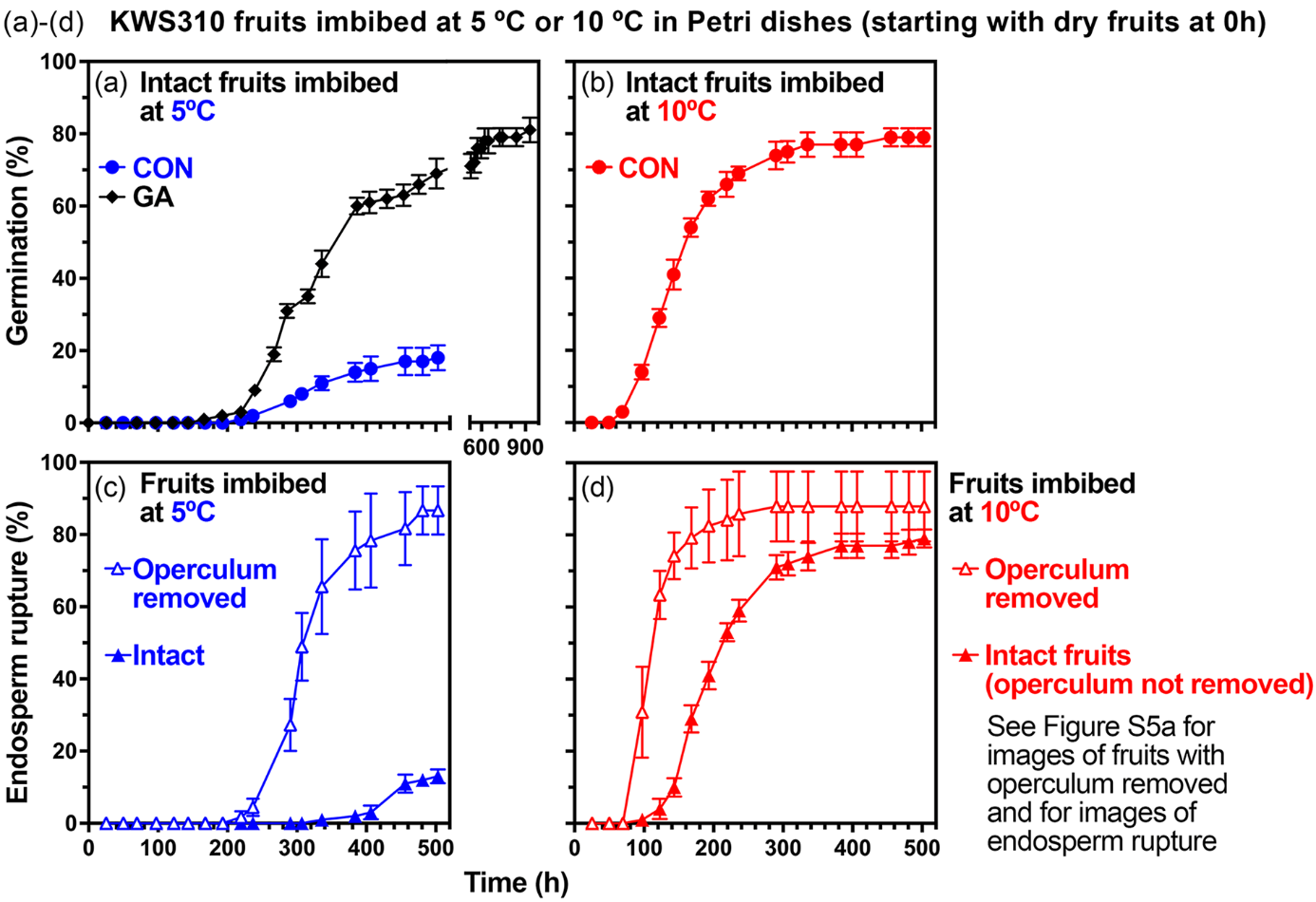
**

**
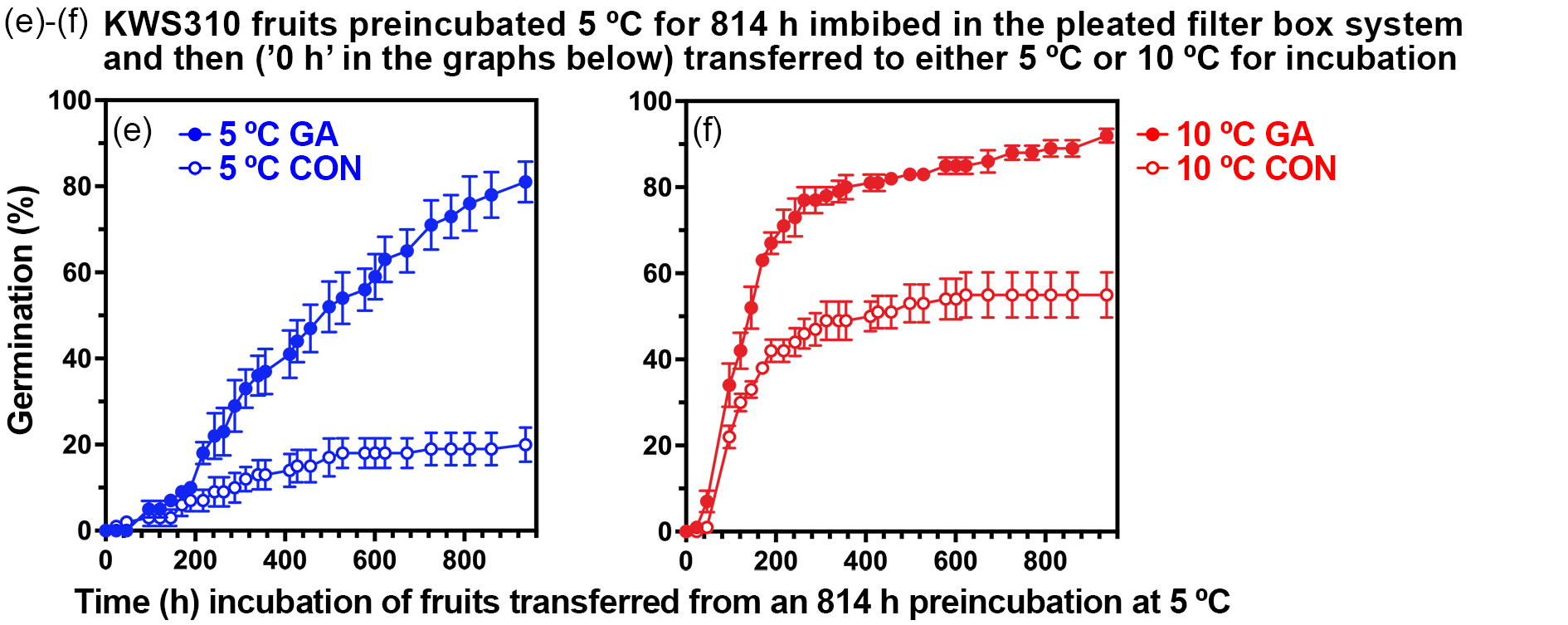
**

**Figure S3.** **Effects of gibberellin and operculum removal on sugar beet germination and secondary dormancy induction of seedlot KWS310.** (a,b) Intact fruits were imbibed at either 5ºC (*left panel*) or 10ºC (*right panel*) without (control, CON) or with 10 µM GA_4+7_ (GA) added. Petri dish system with 2 filter papers and 4 ml water, incubation in darkness, four replicates each with 25 fruits, mean values ± SEM are presented. Fruit germination was scored as the radicle protruding through and beyond the margin of the operculum of the fruit. (c,d) The effect of operculum removal (scarification) on the germination of fruits. The completion of seed germination was scored as endosperm rupture (see Figure S5a). Petri dish system as above. (e,f) The effect of preincubation at 5ºC for 814 h on the physiological dormancy and their release by 10 µM GA_4+7_ (GA) during subsequent incubation at either 5ºC (*left panel*) or 10ºC (*right panel*). Note that compared to the other experiments in the presented work, the KWS310 seedlot used in the experiments presented in this figure (conducted late summer 2021) had lost 15-20% of its seed viability due to the prolonged storage (since 2014). Despite this it had retained its general ability for secondary dormancy induction which was achieved by the 814 h preincubation in white boxes (pleated filter paper system with 30 ml water) in darkness. Subsequent transfer (at '0h' of the x-axes) was to either 5ºC or 10ºC without (CON) or with GA added. Fruit germination was scored as the radicle protruding through and beyond the margin of the operculum of the fruit. Mean values ± SEM are presented of four replicates each with 25 fruits.

**
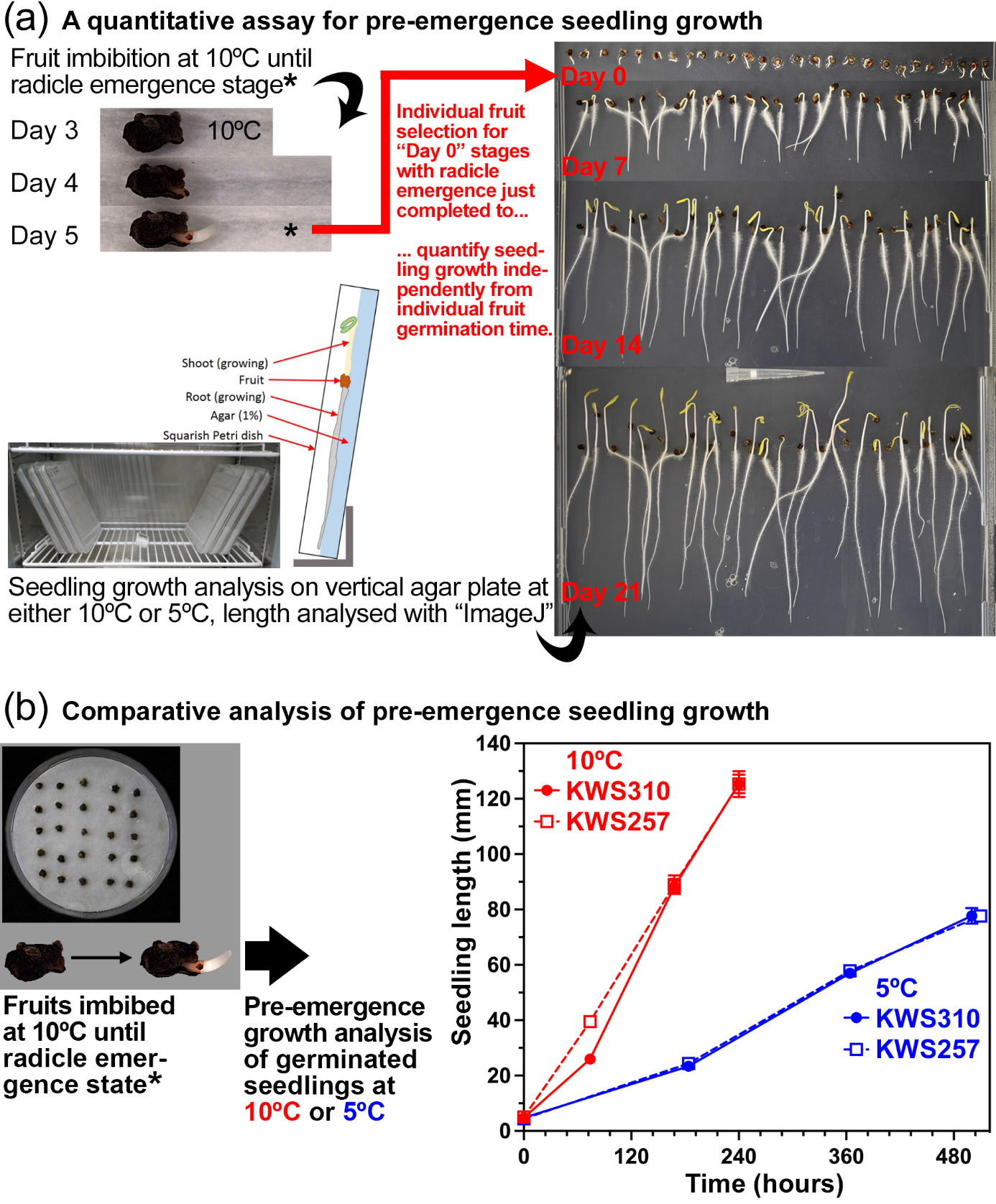
**

**Figure S4. Sugar beet pre-emergence seedling growth analysis.** (a) Pre-emergence seedling growth assay. Fruits were imbibed at 10ºC (standard germination assay conditions) until the radicle emergence stage. This stage with radicle emergence just completed was selected as "day 0" for the pre-emergence seedling growth assay. "Day 0" fruits were transferred to a vertical agar plate system used to monitor seedling growth over time at either 10ºC or 5 º. (b) Comparative analysis of KWS310 and KWS257 seedlings.

**
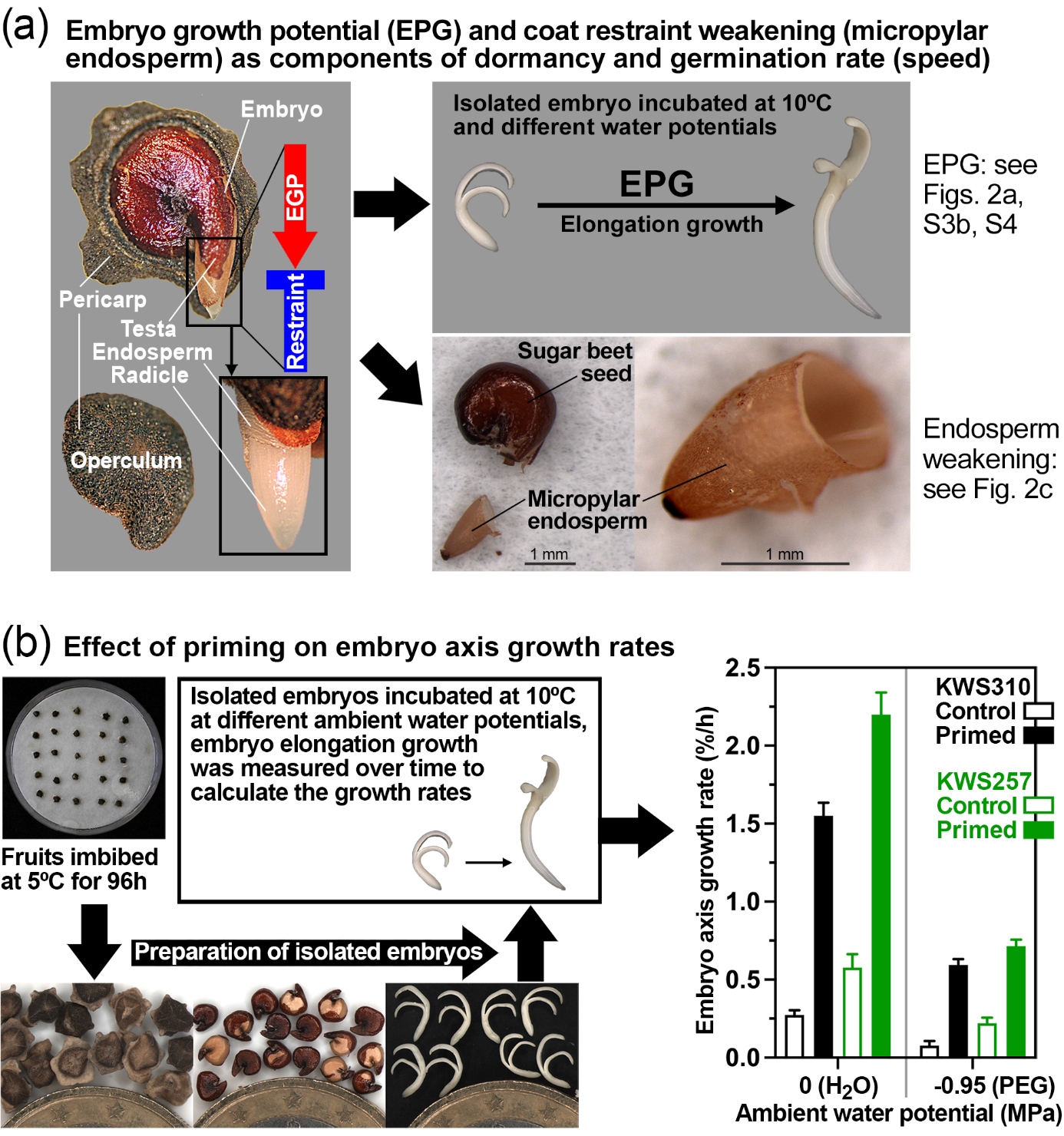
**

**Figure S5.** **Components that define sugar beet dormancy and germination.** (a) Analysis of sugar beet embryo growth potential (EPG) and endosperm restraint weakening. (b) The effect of priming on embryo growth. Priming is known to improve the seed quality of sugar beet (Kockelmann et al., 2011; Lamichhane et al., 2019; Salimi & Boelt, 2019)

**
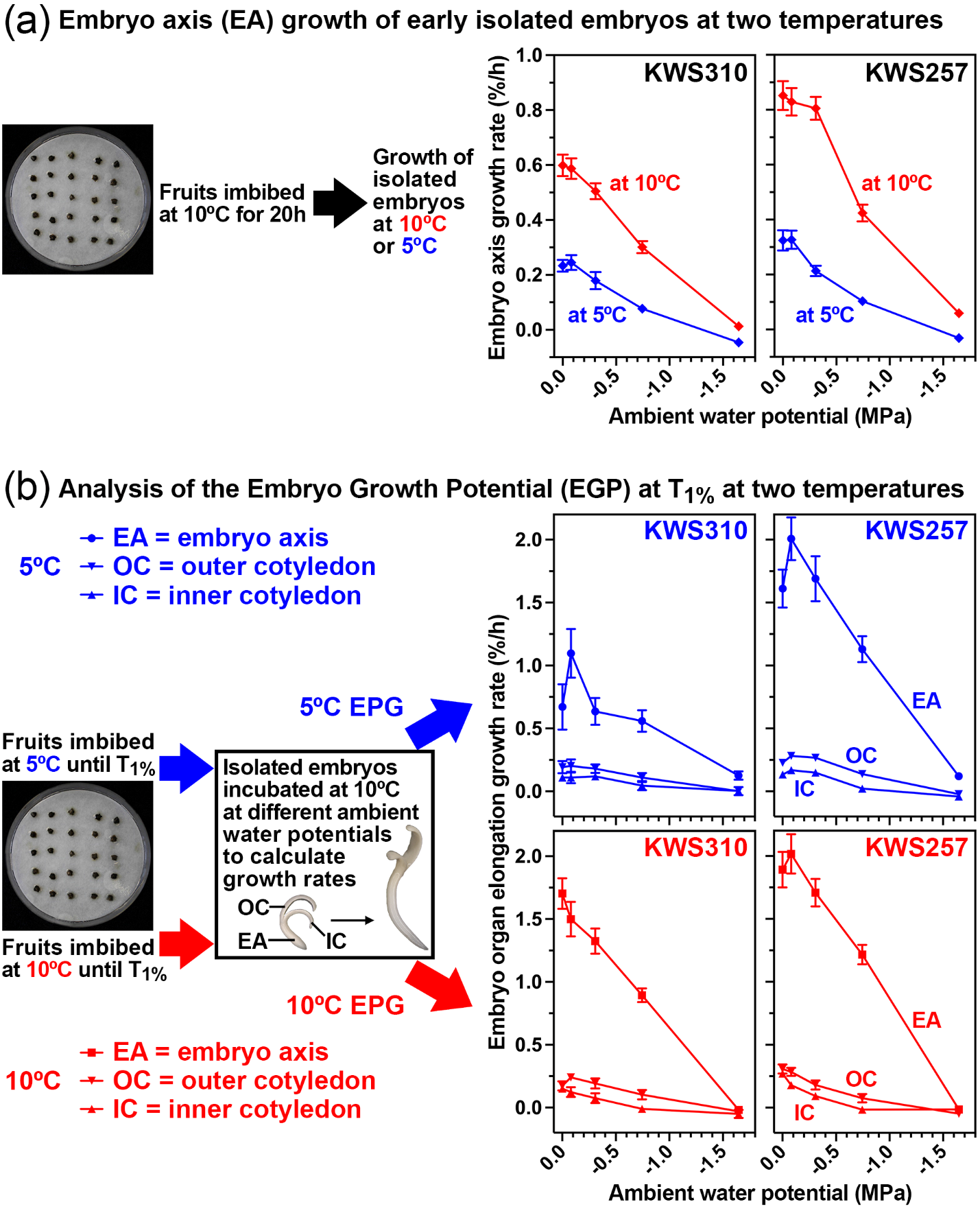
**

**Figure S6.** **Analysis of sugar beet embryo growth potential (EPG).** (a) EPG during early imbibition. (b) EPG at T_1%_. To determine the EPG ungerminated fruits were incubated in white boxes at 5ºC or 10ºC. At the physical or physiological time point whereupon both sets of fruits were at ~1% (T_1%_) of their potential germination the fruits were dissected, the pericarp, testa and perisperm removed, and the embryo used alone. Image J (Schneider et al., 2012) was used to quantify the embryo growth.

**
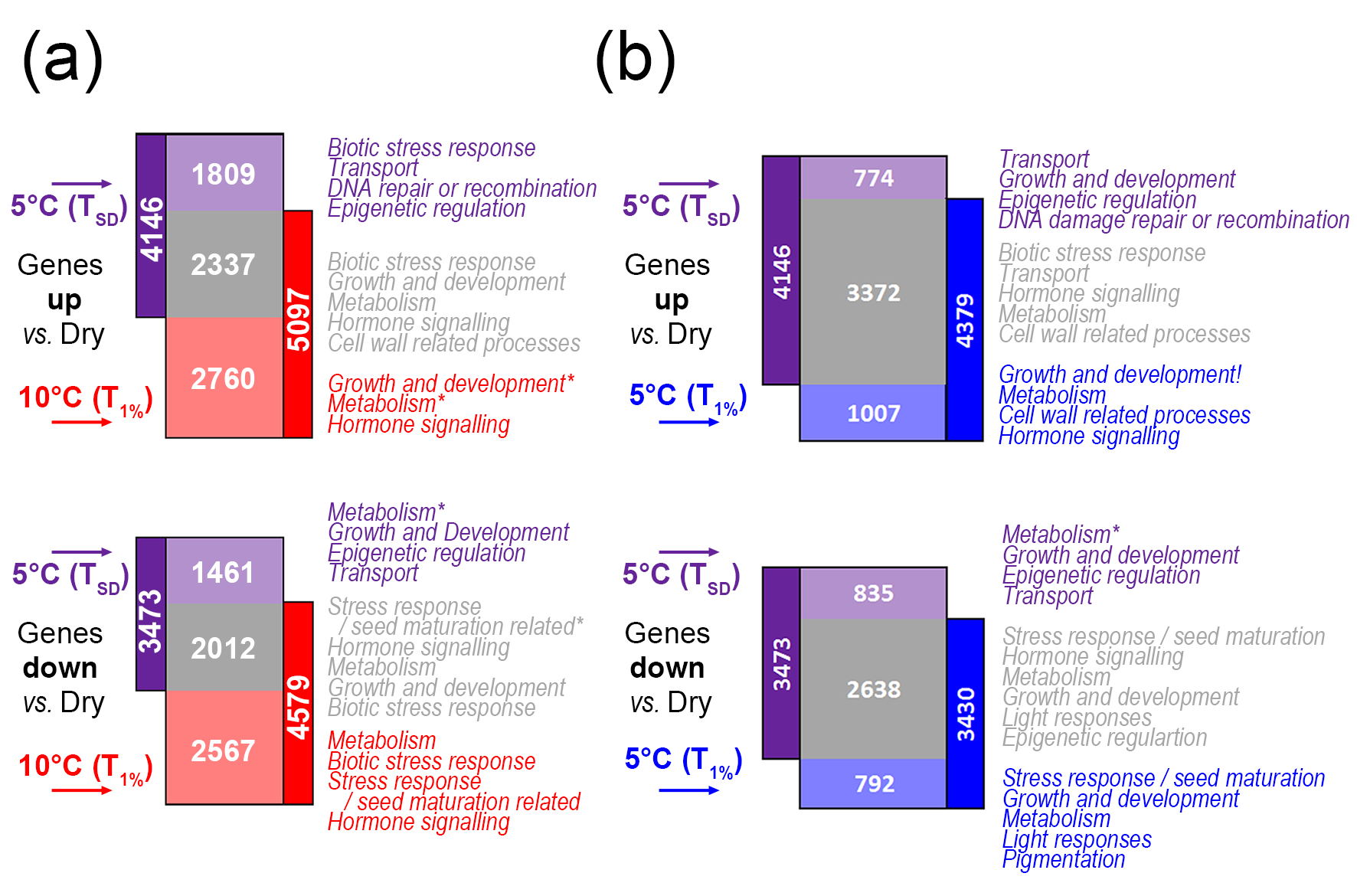
**

**Figure S7.** **Transcriptome analysis of cold-induced secondary dormancy of sugar beet KWS310.** (a) An overview of the number of differentially expressed genes (DEGs) relative to dry fruit up/down at two physiological timepoints, the time at which secondary dormancy is established (T_SD_) under 5ºC and the time point at which 1% of a readily germinating population has germinated (T_1%_). These Venn diagrams highlight the lack of overlap in DEGs in a germinating population at 10ºC and a population that has acquired secondary dormancy at a lower temperature. (b) The number of overlapping and non-overlapping up or down regulated DEGs in fruits relative to dry controls. The physiological timepoints of T_1%_ 5ºC and T_SD_ 5ºC show a large proportion of overlapping DEGs suggesting that the transcriptomic processes required to establish secondary dormancy are initiated early in the germination process. Summary terms that are common in each category are noted next to the relevant bar; * indicates that in this case, the term (e.g. metabolism) is strongly represented, significantly more than other summary terms. See Supplemental Data File 2 for additional DEGs comparisons and details on the GO term analysis.


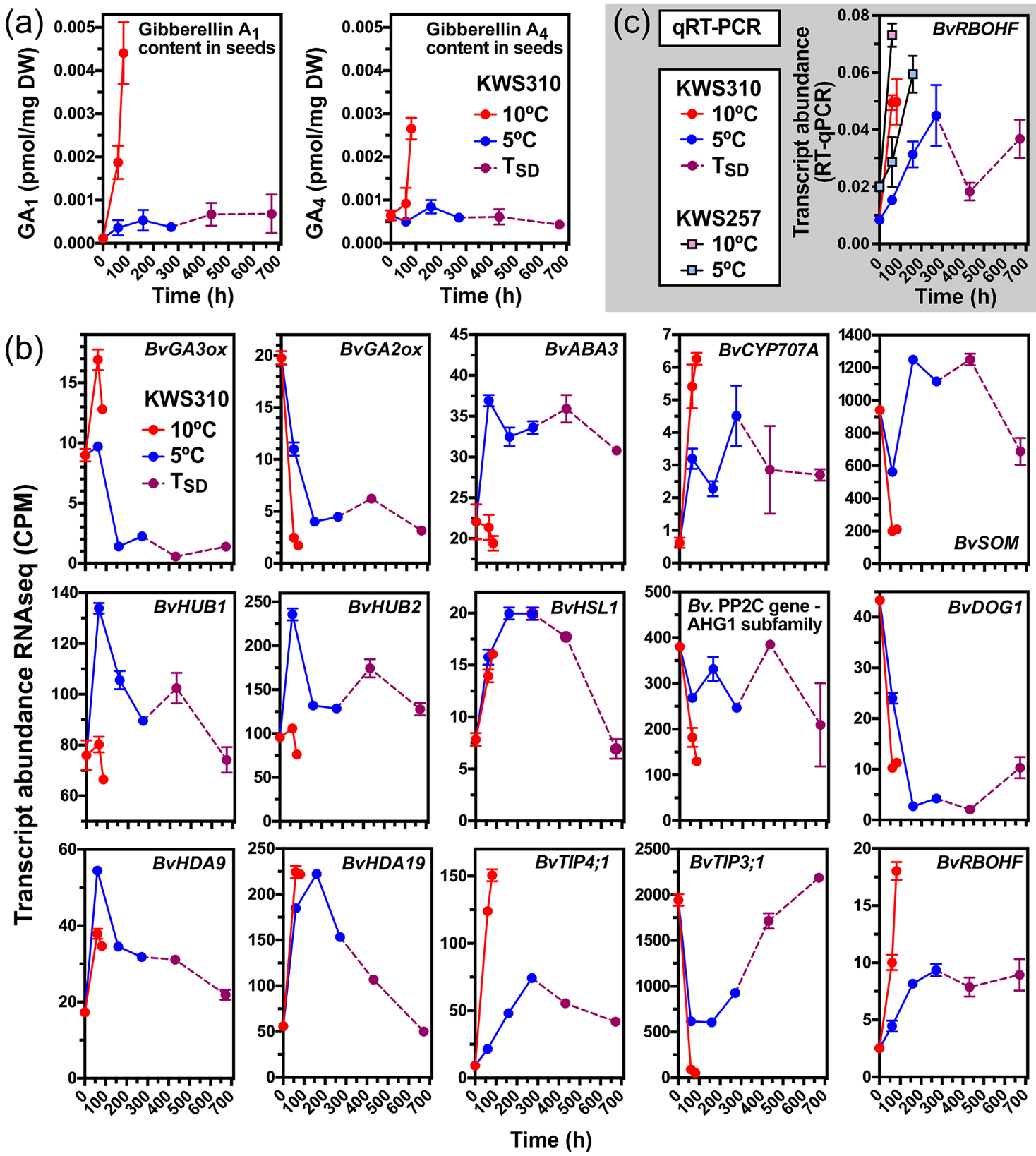


**Figure S8.** **Gibberellin contents, and regulation of genes implicated in the cold-induced secondary dormancy.** (a) Gibberellin A_1_ and gibberellin A_4_ contents in KWS310 sugar beet seeds extracted from incubated fruits at the indicated temperature regimes (n = 5 x 100). (b) Transcript abundances of genes in KWS310 seeds from fruits incubated at the indicated temperature regimes: red (10ºC), blue (5ºC), purple (T_SD_). (c) RT-qPCR comparison of transcript abundances of the contrasting KWS310 and KWS257 seedlots. See Figure 4 for details of the RNAseq and RT-qPCR. Mean values ± SEM are presented.


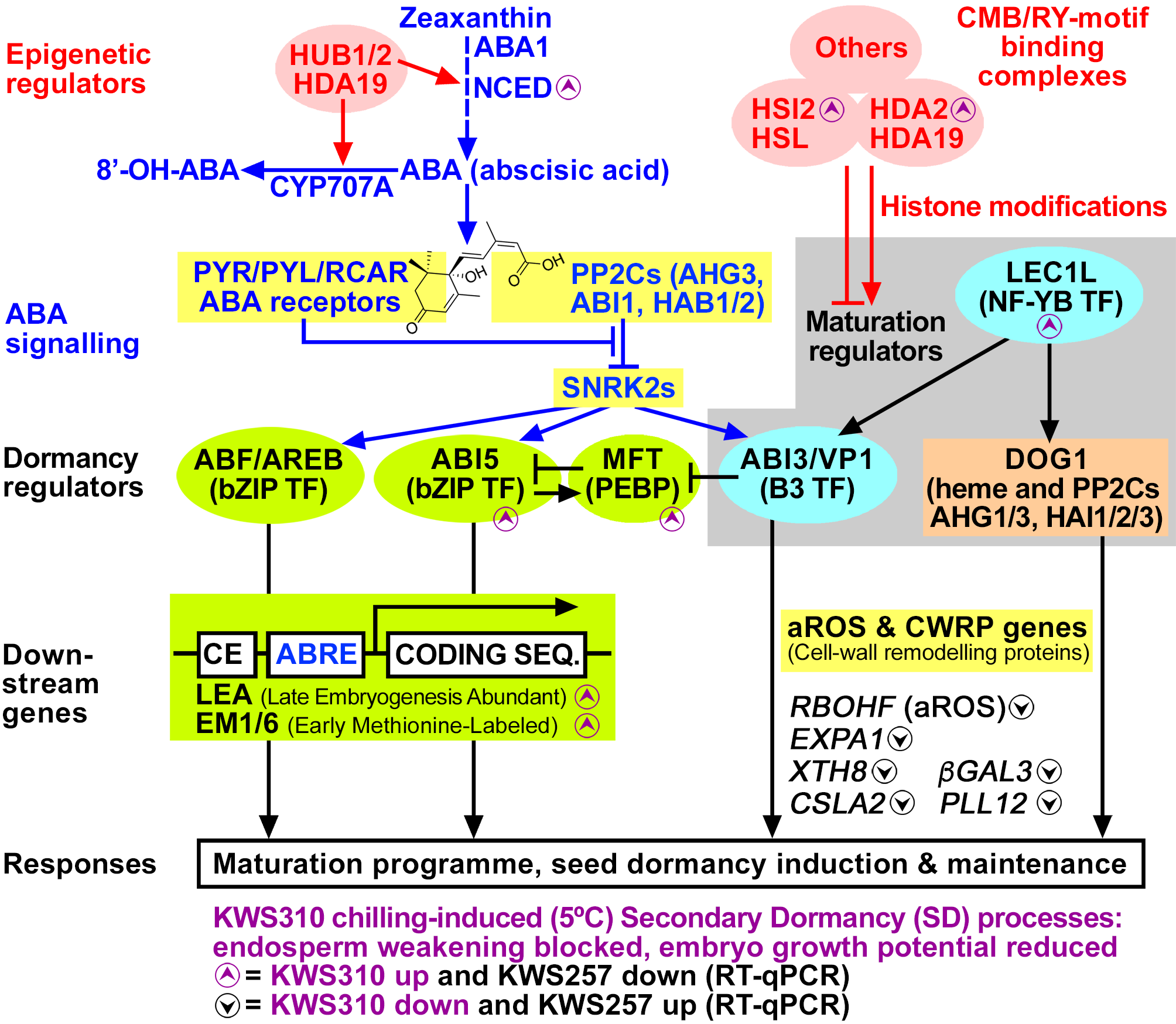


**Figure S9.** **Working model for the epigenetic regulation, signalling and interaction between master regulators of maturation and dormancy implicated in the cold-induced physiological secondary dormancy of sugar beet.** The ABA contents, contrasting expression patterns of seedlots KWS310 and KWS257 at 5ºC (secondary dormancy programme for KWS310; germination programme for KWS257) and 10ºC (germination programme for both), together with the endosperm weakening and germination physiology support the interactions presented in this hypothetical model for the cold-induced dormancy induction. Distinct epigenetic regulation via histone modifications is proposed based on the observed expression differences for many epigenetic regulators (for details, references and abbreviations of epigenetic regulators see the main text, discussion section 4.3). ABA-dependent and independent pathways are known to control maturation and primary dormancy of seeds on the mother plant (Graeber et al., 2012; Hilhorst & Downie, 1995; Leprince et al., 2017). ABA binding to its receptors leads to inhibition of protein phosphatase 2C (PP2C) activity and activation of SNF1-RELATED KINASE 2 (SnRK2) protein kinases, which target downstream components including major dormancy regulators (Bradford et al., 2003; Leprince et al., 2017; Nakashima et al., 2009; Nishimura et al., 2018; Yan et al., 2014). In *Arabidopsis thaliana* these dormancy regulators include bZIP transcription factors (TFs) such as ABA RESPONSIVE ELEMENT-BINDING FACTORs including ABF2 and ABA INSENSITIVE 5 (ABI5), as well as the B3 domain TF ABA INSENSITIVE 3 / Viviparous 1 (ABI3/VP1). The phosphatidylethanolamine-binding protein (PEBP) MOTHER OF FT AND TFL1 (MFT) is known to mediate ABI5-ABI3 and ABA-GA interactions as well as responses to low temperature (Finch-Savage & Footitt, 2017; Graeber et al., 2012; Vaistij et al., 2018). ABF2 and ABI5 have the ABA responsive *cis*-acting elements (ABRE) as target to activate the expression of downstream genes including for LEA and EM1/6 genes (Hundertmark & Hincha, 2008; Leprince et al., 2017). The major seed maturation master regulators of the NF-YB TF subfamily *LEAFY COTYLEDON1* (*LEC1*) and *LEC1-LIKE* (*LEC1L*) bind to the CCAAT box of downstream genes. LEC1 and LEC1L can complement each other to activate transcription by interacting with the seed-specific ABRE-binding TFs (Gnesutta et al., 2017; Jo et al., 2020; Leprince et al., 2017; Wilhelmsson et al., 2019; Yamamoto et al., 2009). These works also revealed combinatory interactions of the LEC1/LEC1L TF with AREB bZIP and the ABI3 TFs which together are required to regulate most of the genes of the maturation programme, including for ABA biosynthesis, LEA and oleosin expression, and other genes of the network. LEC1/LEC1L TF control seed maturation to a major part via activation of ABI3/VP1 and the DELAY OF GERMINATION 1 (DOG1) protein. DOG1 is a master regulator of dormancy and maturation (Bentsink et al., 2006; Bryant et al., 2019; Dekkers et al., 2016) and interacts in complexes with PP2C of the AGH1 subfamily (AGH1, AGH3, HAI1, HAI2, HAI3) via binding to heme (Nee et al., 2017; Nishimura et al., 2018). Downstream apoplastic Reactive Oxygen Species (aROS) and cell-wall remodelling proteins (CWRP) contribute to the induction and maintenance of seed dormancy in response to ambient environmental cues (Finch-Savage & Leubner-Metzger, 2006; Graeber et al., 2014; Graeber et al., 2012; Linkies & Leubner-Metzger, 2012; Müller et al., 2009). The expression patterns of *Beta vulgaris* homologs of these genes (Table S1 and figures of this work) support this framework as a working model for the molecular mechanisms underpinning the regulation of cold-induced physiological secondary dormancy of seedlot KWS310 at 5ºC. Further support for this comes from the RT-qPCR results with the contrasting seedlot KWS257 for which the expression patterns at 5ºC reveal as expected the patterns of the germination programme. While the presented model clearly reveals the key regulatory and downstream components of the two counteracting programmes (physiological secondary dormancy *versus* germination), the temperature-specific triggers of the programme switch remain to be elucidated. Figure 5c show that key candidate genes which may be important for this including from the cold signalling pathway and the epigenetic regulatory system in seeds (Chen et al., 2020; Chhun et al., 2016; Liu et al., 2019; Ma et al., 2015; Nonogaki, 2017).


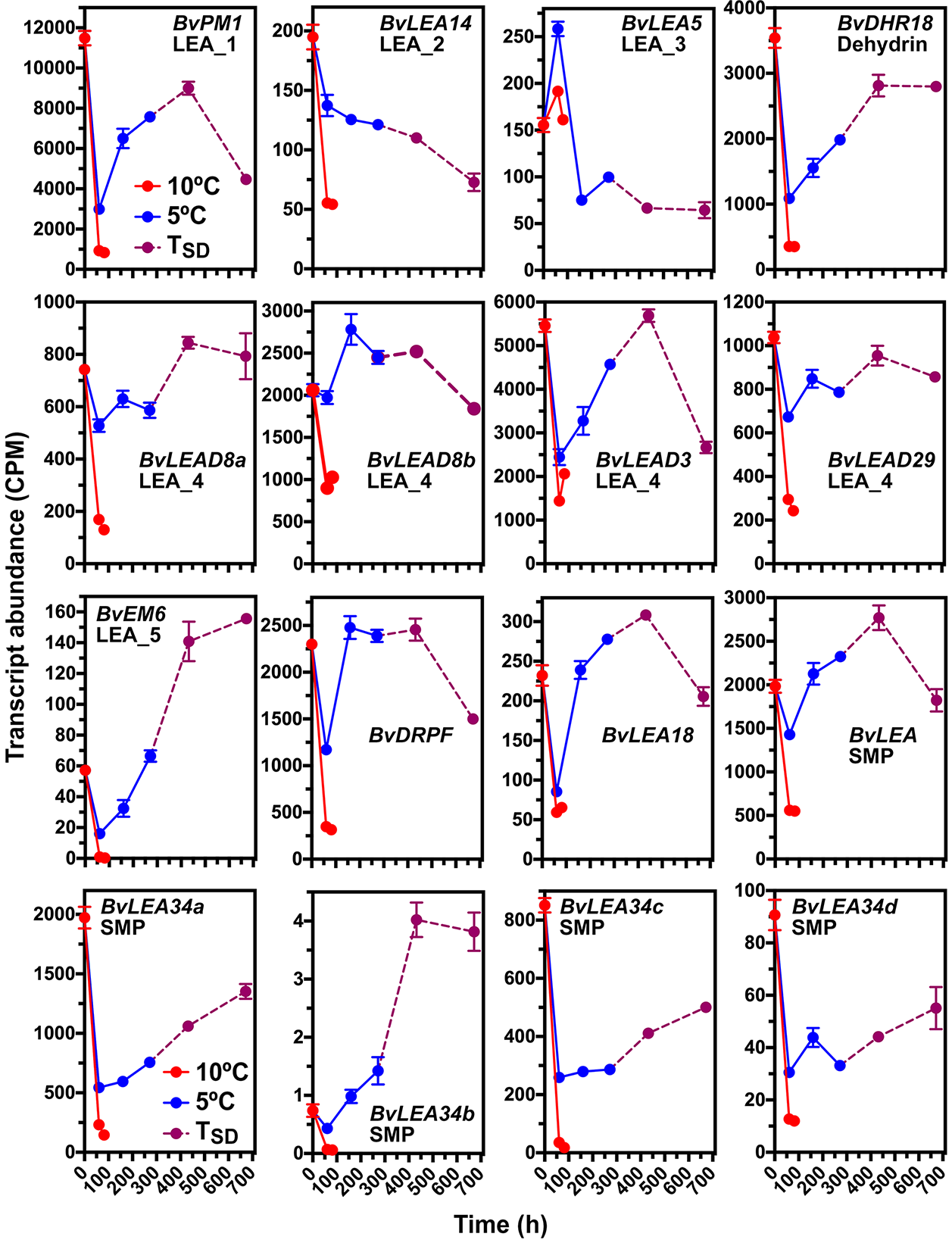


**Figure S10.** **Transcript abundances for late embryogenesis abundant protein genes** **(*BvLEA*) in KWS310 sugar beet seeds** incubated at the indicated temperature regimes: red (10ºC), blue (5ºC), purple (T_SD_), for details of the transcriptome analysis see Figure 2 (n = 3 x 100). See main text for gene abbreviations. The group of the particular LEA gene according to the nomenclature of Hundertmark and Hincha (Hundertmark & Hincha, 2008) is indicated. For gene IDs of individual sugar beet LEA genes see Supplemental Table 1. Mean values ± SEM are presented.

**
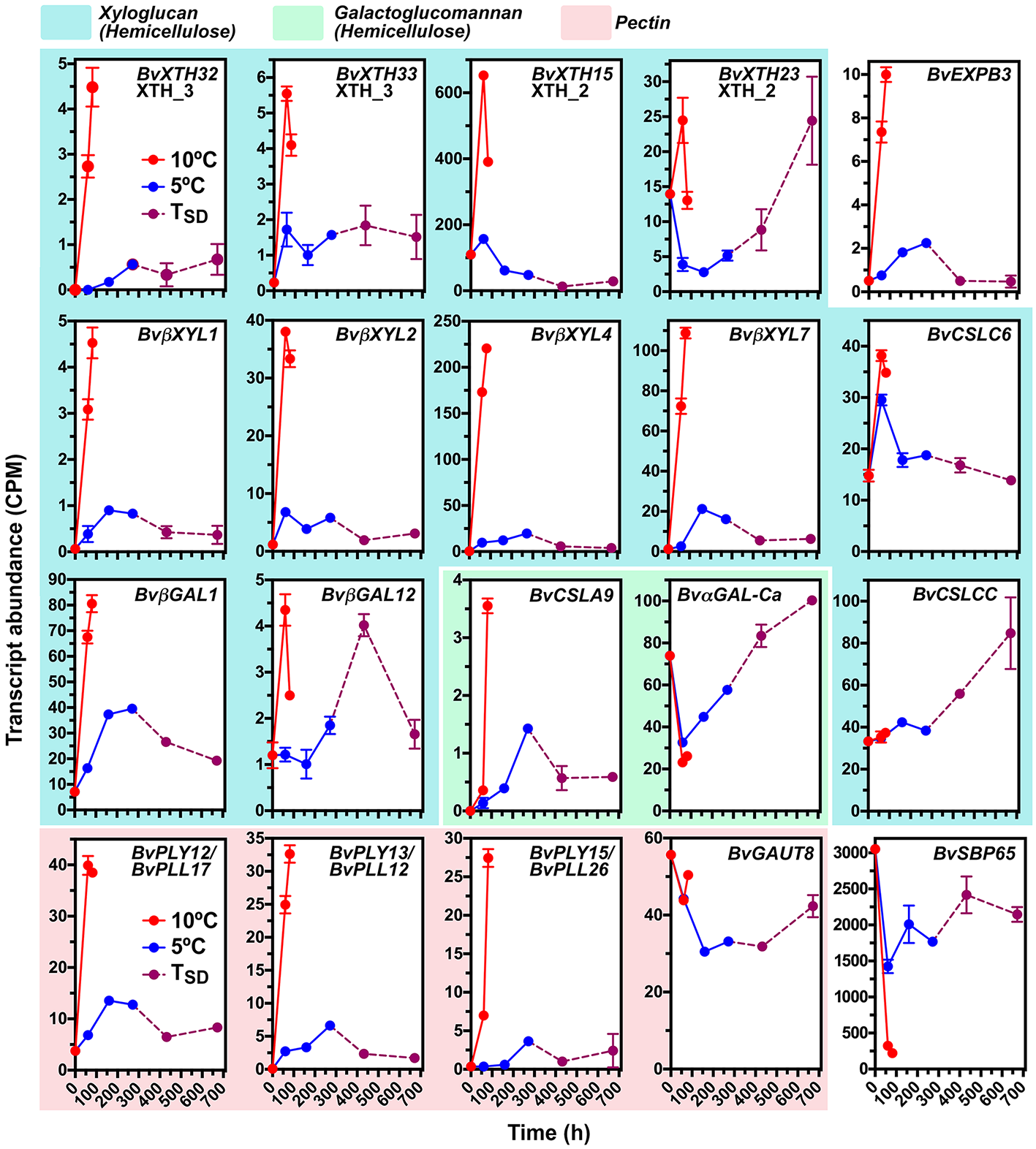
**

**Figure S11.** **Transcript abundances for cell wall remodelling genes in KWS310 sugar beet seeds** incubated at the indicated temperature regimes: red (10ºC), blue (5ºC), purple (T_SD_), for details of the transcriptome analysis see Figure 2 (N = 3 x 100). See main text for gene abbreviations. The group of the particular XTH gene according to the nomenclature of Rose et al (Rose et al., 2002) is indicated. For gene IDs of individual sugar beet genes see Supplemental Table 1. Mean values ± SEM are presented.

Xylosidases are known to play essential roles in xyloglucan remodelling and seed germination in *A. thaliana* (Sechet et al., 2016; Shigeyama et al., 2016). Similar expression patterns compared to XTH cluster 1 (Figure 6a) were observed for other hemicellulose remodelling protein genes, namely α-xylosidase (*BvαXYL1*), several β-xylosidases (*BvβXYL*) and β-galactosidases (*BvβGAL*) which were all upregulated at 10ºC and after a small transient increase at 5ºC, downregulated in association with secondary dormancy (Figures 6c and S10). Cellulose synthase-like C (*BvCSLC*) genes, encoding glycosyltransferases involved in xyloglucan biosynthesis, exhibited distinct expression patterns (Figures 6c and S10), while cellulose synthase 2 (*BvCESA2*) exhibited an expression pattern similar to XTH cluster 2 (Figure 6e). The *mucilage-modified 2 (MUM2)* gene encodes a β-galactosidase and *BvMUM2* was expressed in sugar beet fruits with a pattern similar to XTH cluster 1 (Figure 6d). Cellulose synthase-like A (*CSLA*) genes are involved in the biosynthesis of galactoglucomannans present in the endosperm of many seeds and their mobilisation is achieved by α-galactosidases (*αGAL)* (Rodriguez-Gacio Mdel et al., 2012). *BvCSLA2* and *BvαGAL1* were expressed with patterns similar to XTH cluster 1 (Figures 6f and S10).

**Table S1.** Names and gene IDs of *Beta vulgaris* genes presented in the Figures.

| **Type^a^** | **Figure** | **Name** | **Gene ID** |
| --- | --- | --- | --- |
| **Single genes** | **4** | *BvZEP* / *BvABA1* | Bv_003210_fkxn.t1 |
|  |  | *BvNCEDa* | Bv4_094240_texx.t1 |
|  |  | *BvNCEDb* | Bv9_204380_sxfy.t1 |
|  |  | *BvCYP707A2* | Bv7_173550_exhw.t1 |
|  |  | *BvABI3* | Bv7_167370_hxew.t1 |
|  |  | *BvABI5* | Bv7_159570_afnu.t1 |
|  |  | *BvMFT* | Bv8_181360_amkr.t1 |
|  |  | *BvABF2* | Bv7_169340_mcdu.t1 |
|  | **5** | *BvLEC1L* | Bv8_184360_ryex.t1 |
|  |  | *BvLEA34a* | Bv3_068240_ksfm.t1 |
|  |  | *BvEM6* | Bv5_105820_ddrx.t1 |
|  |  | *BvEM1* | Bv5_102930_zweh.t1 |
|  |  | *BvCOLD1-G* | Bv3_056050_fyzs.t1 |
|  |  | *BvCOR413-TM2* | Bv_003940_ujja.t1 |
|  |  | *BvCOR413-PM4* | Bv6_137990_igpa.t1 |
|  |  | *BvHSI2* | Bv7_175800_ycqe.t1 |
|  |  | *BvHDA2* | Bv_006940_mxka.t1 |
|  |  | *BvTIP1;3* / *BvCOLD1* | Bv7_179210_kzkq.t1 |
|  |  | *BvMYB44* | Bv6_127480_such.t1 |
|  |  | *BvMYB62* | Bv6_141520_usyi.t1 |
|  |  | *BvCBF5* | Bv7_174020_ttaz.t1 |
|  |  | *BvACO1* | Bv_008110_nwgk.t1 |
|  |  | *BvACO2/4* | Bv9_204220_gdtf.t1 |
|  | **6** | *BvEXPA1* | Bv9_215900_jwjj.t1 |
|  |  | *BvXTH8* | Bv8_200100_otoo.t1 |
|  |  | *BvXTH16* | Bv3_065220_jynp.t1 |
|  |  | *BvαXYL1* | Bv6_142010_npsj.t1 |
|  |  | *BvCSLC5* | Bv7_168770_skhg.t1 |
|  |  | *BvßGAL3* | Bv6_129600_iugy.t1 |
|  |  | *BvßGAL6* / *BvMUM2* | Bv4_078180_xgtz.t1 |
|  |  | *BvCESA2* | Bv5_116100_sjxf.t1 |
|  |  | *BvCSLA2* | Bv9_211030_encd.t1 |
|  |  | *BvαGAL1* | Bv2_034670_ffcc.t1 |
|  |  | *BvPLY5* /*BvPLL16* | Bv6_147440_tgxc.t1 |
|  | **S8** | *BvGA3ox* | Bv9_220330_apay.t1 |
|  |  | *BvGA2ox* | Bv5_122520_ndnw.t1 |
|  |  | *BvABA3* | Bv9_219130_pnip.t1 |
|  |  | *BvCYP707A* | Bv5_102360_eoic.t1 |
|  |  | *BvSOM* | Bv9_205240_sder.t1 |
|  |  | *BvHUB1* | Bv1_018250_xqjm.t1 |
|  |  | *BvHUB2* | Bv1_008490_gruo.t1 |
|  |  | *BvHSL1* | Bv7_157000_dced.t1 |
|  |  | *BvPP2C* gene *AHG1* subfamily | Bv2_025410_kjqj.t1 |
|  |  | *BvDOG1* | Bv4_078280_ymnd.t1 |
|  |  | *BvHDA9* | Bv2_044990_wqan.t1 |
|  |  | *BvHDA19* | Bv9_218010_wffu.t1 |
|  |  | *BvTIP4;1* | Bv2_032220_ydno.t1 |
|  |  | *BvTIP3;1* | Bv8_188920_dreg.t1 |
|  |  | *BvRBOHF* | Bv5_099740_zpio.t1 |
|  | **S10** | *BvPM1* | Bv3_060300_dgcf.t1 |
|  |  | *BvLEA14* | Bv1_003950_hwof.t1 |
|  |  | *BvLEA5* | Bv7_163660_qugz.t1 |
|  |  | *BvDHR18* | Bv6_129380_msqq.t1 |
|  |  | *BvLEAD8a* | Bv8_183650_ietu.t1 |
|  |  | *BvLEAD8b* | Bv7_160770_ufkf.t1 |
|  |  | *BvLEAD3* | Bv9_220110_fpuf.t1 |
|  |  | *BvLEAD29* | Bv1_017290_dyfj.t1 |
|  |  | *BvEM6* | Bv2_023570_xxaq.t1 |
|  |  | *BvDRPF* | Bv8_182890_ghwm.t1 |
|  |  | *BvLEA18* | Bv6_127730_chca.t1 |
|  |  | *BvLEA* | Bv5_106150_qehe.t1 |
|  |  | *BvLEA34a* | Bv3_068240_ksfm.t1 |
|  |  | *BvLEA34b* | Bv4_082430_pdep.t1 |
|  |  | *BvLEA34c* | Bv5_119390_rinm.t1 |
|  |  | *BvLEA34d* | Bv5_119410_ofut.t1 |
|  | **S11** | *BvXTH32* | Bv9_205660_jhqe.t1 |
|  |  | *BvXTH33* | Bv6_149030_cndy.t1 |
|  |  | *BvXTH15* | Bv3_065230_efuk.t1 |
|  |  | *BvXTH23* | Bv5_110620_yqwe.t1 |
|  |  | *BvXEXPB3* | Bv9_206880_rowz.t1 |
|  |  | *BvßXYL1* | Bv3_053740_wtqc.t1 |
|  |  | *BvßXYL2* | Bv5_121630_wrwg.t1 |
|  |  | *BvßXYL4* | Bv8_190250_nxyi.t1 |
|  |  | *BvßXYL7* | Bv1_009530_rznm.t1 |
|  |  | *BvCSLC6* | Bv7_168230_qydw.t1 |
|  |  | *BvßGAL1* | Bv7_176740_serx.t1 |
|  |  | *BvßGAL12* | Bv_007030_wter.t1 |
|  |  | *BvCSLA9* | Bv4_093860_etgh.t1 |
|  |  | *Bv*α*GAL-Ca* | Bv6_146990_iuqd.t1 |
|  |  | *BvCSLCC* | Bv1_014610_ygyy.t1 |
|  |  | *BvPLY12* / *BvPLL17* | Bv3_069240_kufg.t1 |
|  |  | *BvPLY13* / *BvPLL12* | Bv7_157420_nccy.t1 |
|  |  | *BvPLY15* / *BvPLL26* | Bv3_056470_akqt.t1 |
|  |  | *BvGAUT8* | Bv7_178850_ydce.t1 |
|  |  | *BvSBP65* | Bv9_225180_ungj.t1 |

| **Gene lists** | **5** | *BvLEAs* | Bv1_003950_hwof.t1 |
| --- | --- | --- | --- |
|  |  |  | Bv7_163660_qugz.t1 |
|  |  |  | Bv4_082430_pdep.t1 |
|  |  |  | Bv5_119400_etzo.t1 |
|  |  |  | Bv3_068240_ksfm.t1 |
|  |  |  | Bv5_119420_raqo.t1 |
|  |  |  | Bv5_119410_ofut.t1 |
|  |  |  | Bv5_119390_rinm.t1 |
|  |  |  | Bv6_129380_msqq.t1 |
|  |  |  | Bv5_106150_qehe.t1 |
|  |  |  | Bv9_220110_fpuf.t1 |
|  |  |  | Bv2_025370_ughh.t1 |
|  |  |  | Bv7_160770_ufkf.t1 |
|  |  |  | Bv8_182890_ghwm.t1 |
|  |  |  | Bv8_183650_ietu.t1 |
|  |  |  | Bv1_001480_uati.t1 |
|  |  |  | Bv1_017290_dyfj.t1 |
|  |  |  | Bv6_127730_chca.t1 |
|  |  |  | Bv2_023570_xxaq.t1 |
|  |  |  | Bv5_102930_zweh.t1 |
|  |  |  | Bv5_105820_ddrx.t1 |
|  |  |  | Bv9_225180_ungj.t1 |
|  |  |  | Bv3_060300_dgcf.t1 |
|  |  | *BvOLEs* | Bv2_024090_pghd.t1 |
|  |  |  | Bv2_032920_more.t1 |
|  |  |  | Bv3_068090_nzrr.t1 |
|  |  |  | Bv4_091150_ypka.t1 |
|  |  |  | Bv5_117750_ncem.t1 |
|  |  |  | Bv5_126030_saqg.t1 |
|  |  | *BvACOs* | Bv_008110_nwgk.t1 |
|  |  |  | Bv9_204220_gdtf.t1 |
|  |  |  | Bv8_185300_wndh.t1 |
|  |  |  | Bv1_010100_oeux.t1 |
|  | **6** | *BvXTH* cluster 1 | Bv8_200100_otoo.t1 |
|  |  |  | Bv_013300_ndyt.t1 |
|  |  |  | Bv6_149030_cndy.t1 |
|  |  |  | Bv_013510_xztt.t1 |
|  |  |  | Bv7_178970_hwnc.t1 |
|  |  |  | Bv9_205660_jhqe.t1 |
|  |  | *BvXTH* cluster 2 | Bv2_040540_duix.t1 |
|  |  |  | Bv5_110610_ydci.t1 |
|  |  |  | Bv5_110620_yqwe.t1 |
|  |  |  | Bv2_040530_tyiu.t1 |
|  |  |  | Bv_013490_psrc.t1 |
|  |  |  | Bv3_065250_doxn.t1 |
|  |  | *BvEXP* | Bv2_042540_wkwn.t1 |
|  |  |  | Bv5_114530_ydgy.t1 |
|  |  |  | Bv2_042560_jxah.t1 |
|  |  |  | Bv2_024950_ujqs.t1 |
|  |  |  | Bv7_173770_onmk.t1 |
|  |  |  | Bv5_110180_yyjz.t1 |
|  |  |  | Bv6_153620_koaq.t1 |
|  |  |  | Bv9_215900_jwjj.t1 |
|  |  |  | Bv9_206880_rowz.t1 |
|  |  | *BvßXYL* | Bv2_047360_scgp.t1 |
|  |  |  | Bv3_053740_wtqc.t1 |
|  |  |  | Bv5_121630_wrwg.t1 |
|  |  |  | Bv6_128220_hjig.t1 |
|  |  |  | Bv6_128220_hjig.t2 |
|  |  |  | Bv6_152900_aoak.t1 |
|  |  |  | Bv8_189590_igsy.t1 |
|  |  |  | Bv8_190250_nxyi.t1 |
|  |  |  | Bv1_009520_fpuu.t1 |
|  |  |  | Bv1_009530_rznm.t1 |
|  |  | *BvPLY* / *BvPLL* | Bv3_069240_kufg.t1 |
|  |  |  | Bv7_157420_nccy.t1 |
|  |  |  | Bv3_056470_akqt.t1 |
|  |  |  | Bv6_147440_tgxc.t1 |
|  |  |  | Bv7_167180_snhs.t1 |
|  |  |  | Bv7_167220_ooua.t1 |
|  |  |  | Bv7_176940_irqf.t1 |
|  |  |  | Bv6_154540_uozn.t1 |
|  |  |  | Bv7_171650_znyz.t1 |
|  |  |  | Bv7_179150_aspd.t1 |
|  |  |  | Bv3_049770_iiws.t1 |
|  |  |  | Bv2_040390_fynm.t1 |
|  |  |  | Bv5_115140_ezoj.t1 |

**^a^** Genes (single genes) plotted in Figures 2, 4, 5, 6, S8, S10 and S11 and Gene IDs of genes belonging to categories (gene lists) whose average expression was plotted in Figures 4, 5 and 6. Transcript expression profiles and details about individual genes are accessible via the Gene Expression Viewer.

**Table S2.** List of primers used for quantitative RT-PCR analysis.

| ***Beta vulgaris* ID** | ***Gene name*** | ***Primer direction*** | **Primer Sequence (5' – 3')** |
| --- | --- | --- | --- |
| Bv4_087220_gudo.t1 | *BvMGL^a^* | F | TCACCTTCCATTTCTCCTCGC |
|  |  | R | CCCATATCCAGTGCACCCAG |
| Bv9_216880_hkhd.t1 | *BvVHA-A3^a^* | F | TGTGACTGATCCTGCTTCTGG |
|  |  | R | TCCTCAGCTCCGAAAGTTTACC |
| Bv7_159570_afnu.t1 | *BvABI5* | F | CGCCAGGTCTAGAGCAAGG |
|  |  | R | CTTCGACTCCTCCTCATCGC |
| Bv9_211030_encd.t1 | *BvCSLA2* | F | CGGGCGGGTCAAATAAGTTC |
|  |  | R | ATCACCATACGATCTGCCGG |
| Bv5_105820_ddrx.t1 | *BvEM6* | F | GAGGTACCGGTGGAAAGAGC |
|  |  | R | GACCTCCTTTACGGCCCATC |
| Bv9_215900_jwjj.t1 | *BvEXPA1* | F | GTGTAATTTCCCTCGCCAGC |
|  |  | R | TCACCGTAAATCTCATCCCACC |
| Bv_006940_mxka.t1 | *BvHDA2* | F | CTCTTCATCACCTGCCTCTCC |
|  |  | R | TGCGACCCCATTTTGAAGAATC |
| Bv7_175800_ycqe.t1 | *BvHSI2* | F | GGTGAAATTGCCGATCTGTGC |
|  |  | R | TCTCTCCAGCCAGTCTCCTC |
| Bv3_068240_ksfm.t1 | *BvLEA34a* | F | AGAGACTGATGTTCCTGGTGC |
|  |  | R | TCGCCTATGGTTACCTTGCC |
| Bv8_184360_ryex.t1 | *BvLEC1L* | F | GCAAGATTCTTCCACCCCATG |
|  |  | R | TGGTCTTACGTTGCTCCCTC |
| Bv8_181360_amkr.t1 | *BvMFT* | F | GGTGCGAAATCAAACCTTCCC |
|  |  | R | TTCGGCTCACTTGGACTTGG |
| Bv9_204380_sxfy.t1 | *BvNCEDb* | F | GCCGCCTCCAAATTACCAAC |
|  |  | R | AACCGTGGACTCTTGAACCG |
| Bv7_157420_nccy.t1 | *BvPLL12* | F | TCACTCTCAATGCTGGCGTC |
|  |  | R | ACCTCCAGTGCCATACATGC |
| Bv5_099740_zpio.t1 | *BvRBOHF* | F | TCTCGTCTCCAGATCTTCTTCG |
|  |  | R | ACTTCTTCTTCGGTCAGGCG |
| Bv8_200100_otoo.t1 | *BvXTH8* | F | GGGGAACAGAACAGGAGAACC |
|  |  | R | GGAAGAAGTCGTTGGGTTTGC |
| Bv6_129600_iugy.t1 | *Bv*$\beta$*GAL3* | F | GGATCGCCCGACACTCATAG |
|  |  | R | TCCTCCCACATTCGGTAAACC |

**^a^** Reference genes: *BvMGL* (putative methionine gamma-lyase) and *BvVHA-*A3 (vacuolar proton ATPase A3 subunit) for the RT-qPCR analyses.

**Supplemental Instructions for Gene Expression Viewer**

***Purpose***

This Excel file allows the user to visualize expression of individual genes of interest or summarised expression of lists of genes of interest in the *Beta vulgaris* cold-induced secondary dormancy transcriptome dataset.

The viewer (main sheet, ‘Viewer’) can display the sum, mean or mean z-score expression summaries for lists of genes and the mean cpm (counts per million mapped reads) of individual genes plotted as a time course for fruits imbibed at 5°C and 10°C. Details of gene lists can be seen in a separate sheet (‘View List’).

Genes / lists of genes plotted in the publication are included as pre-set options. The plots allow viewing of the individual genes of which the gene lists are comprised. Additionally, the file allows for the user to input either their own single gene of interest or lists of genes of interests.

***Password for sheet protection: %sugarbeet***

***Viewing pre-set genes from the publication figures***

***
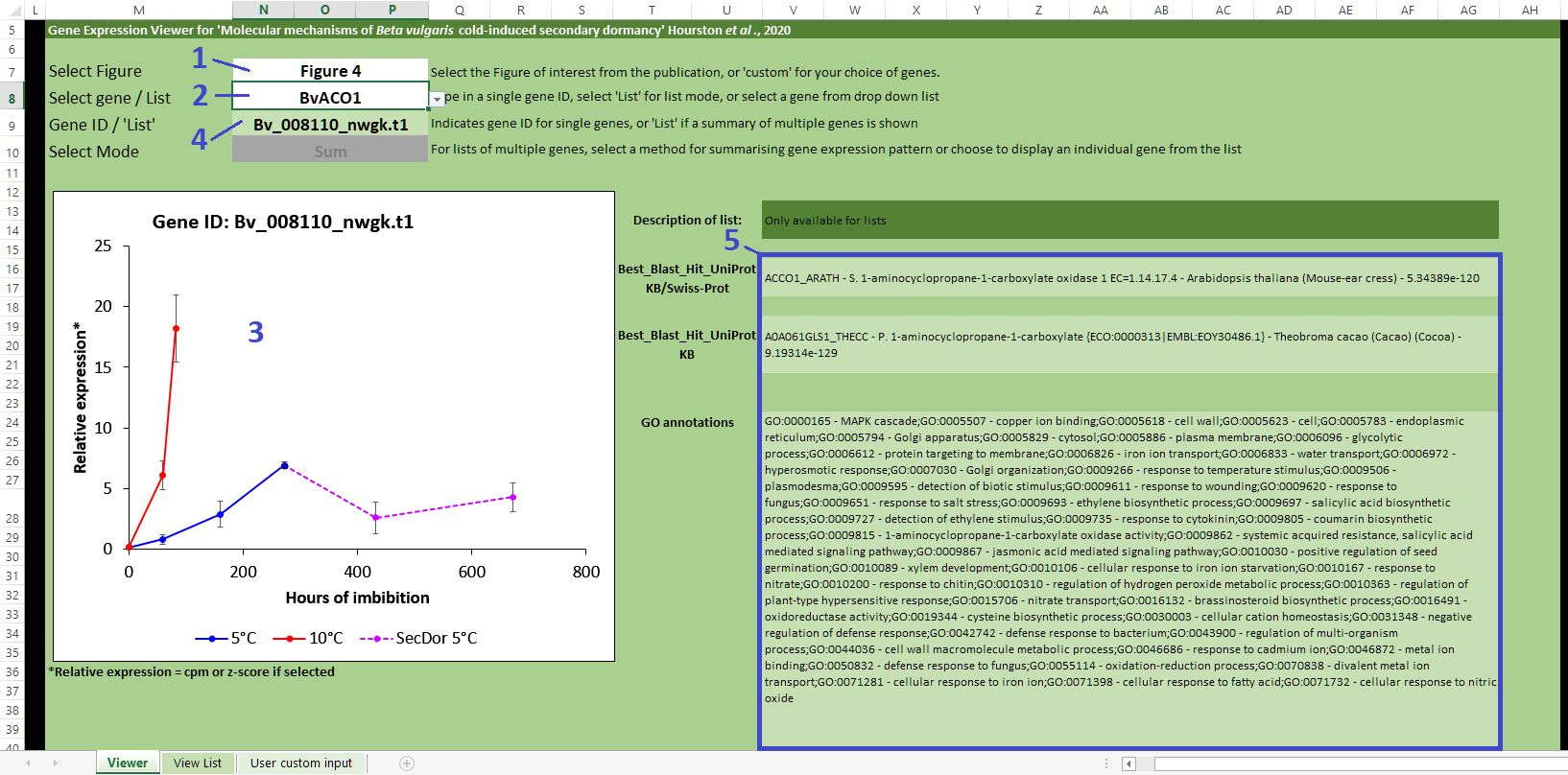
***

For example, to view *BvACO1* from Figure 4 C, select ‘Figure 4’ from the ‘Select Figure’ drop-down list (1)*.* Then select ‘*BvACO1*’ from the ‘Select gene / List’ drop-down list (2). You may have to scroll down the list to find ‘*BvACO1*’. The graph will now display the mean cpm (± standard error) expression time-course for the experiment (3). Additionally, the gene ID for *BvACO1* (4) and associated annotations (5) for the gene will be displayed. ‘Select Mode’ is greyed out as this is only applicable for lists.

***Viewing pre-set gene lists from the publication figures***

**
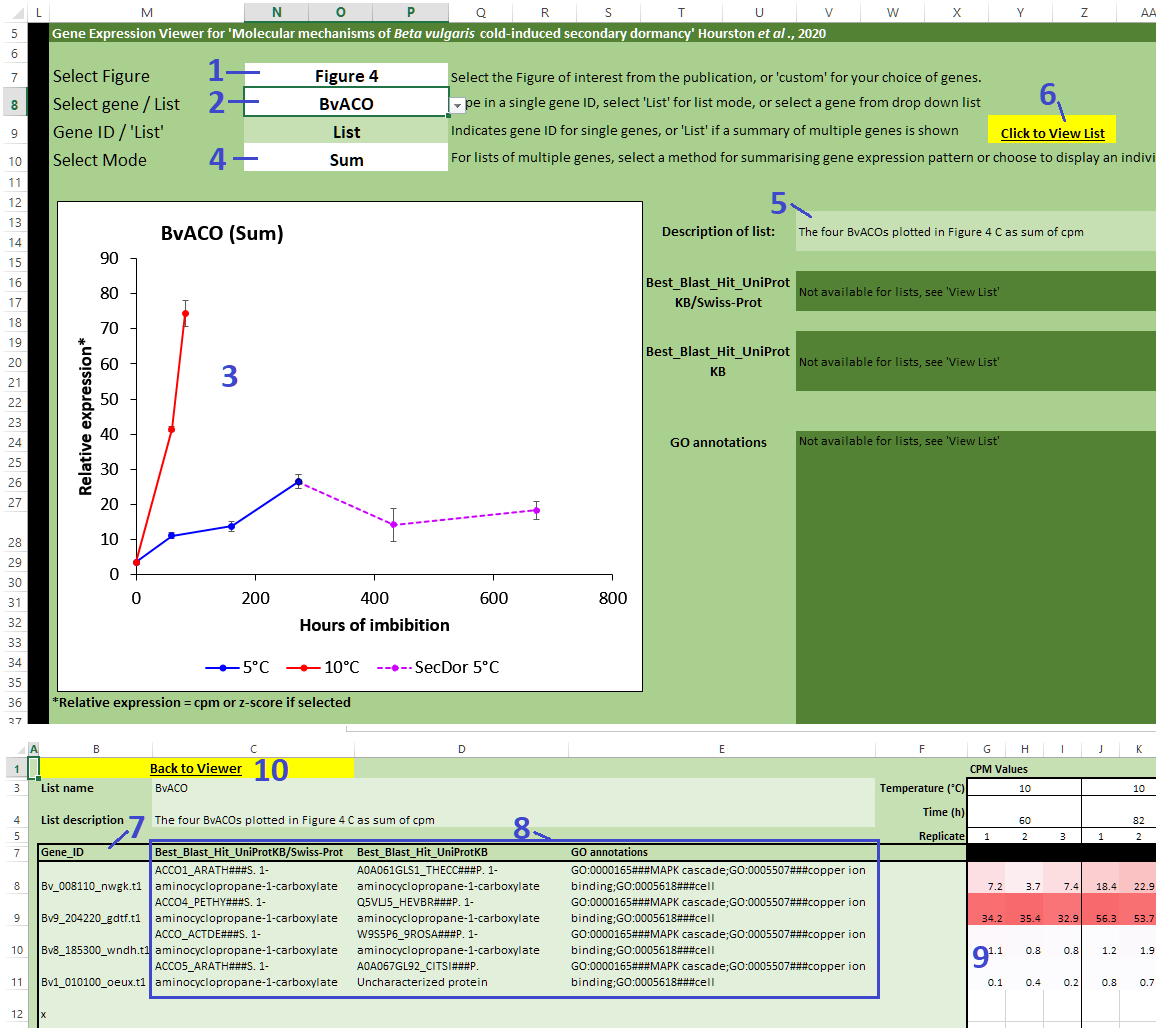
**

The sum expression of all *BvACO* genes is also plotted in Figure 4 C. To view this summary select Figure 4 from ‘Select Figure’ (1), and then ‘*BvACO*’ from *‘*Select gene / List’(2). The graph should now display the sum of cpm (± standard error) for the four *BvACO*s which make up the *BvACO* list (3). The type of summary can be changed by selecting ‘Sum’, ‘Average’ (the mean) or ‘z-score’ (mean of the z-score normalised gene expression) from the ‘Select Mode’ list (4). A description of the list is shown (5). To view the genes in the list click ‘Click to View List’ (6). This page displays the IDs of the genes in the list (7) and associated annotations (8) and cpm expression in each sample (9). The sheet has to be unlocked to copy and paste values and show full length annotations. To return to the main viewer page, click ‘Back to Viewer’ (10).

***Viewing individual genes from pre-set gene lists from the publication figures***


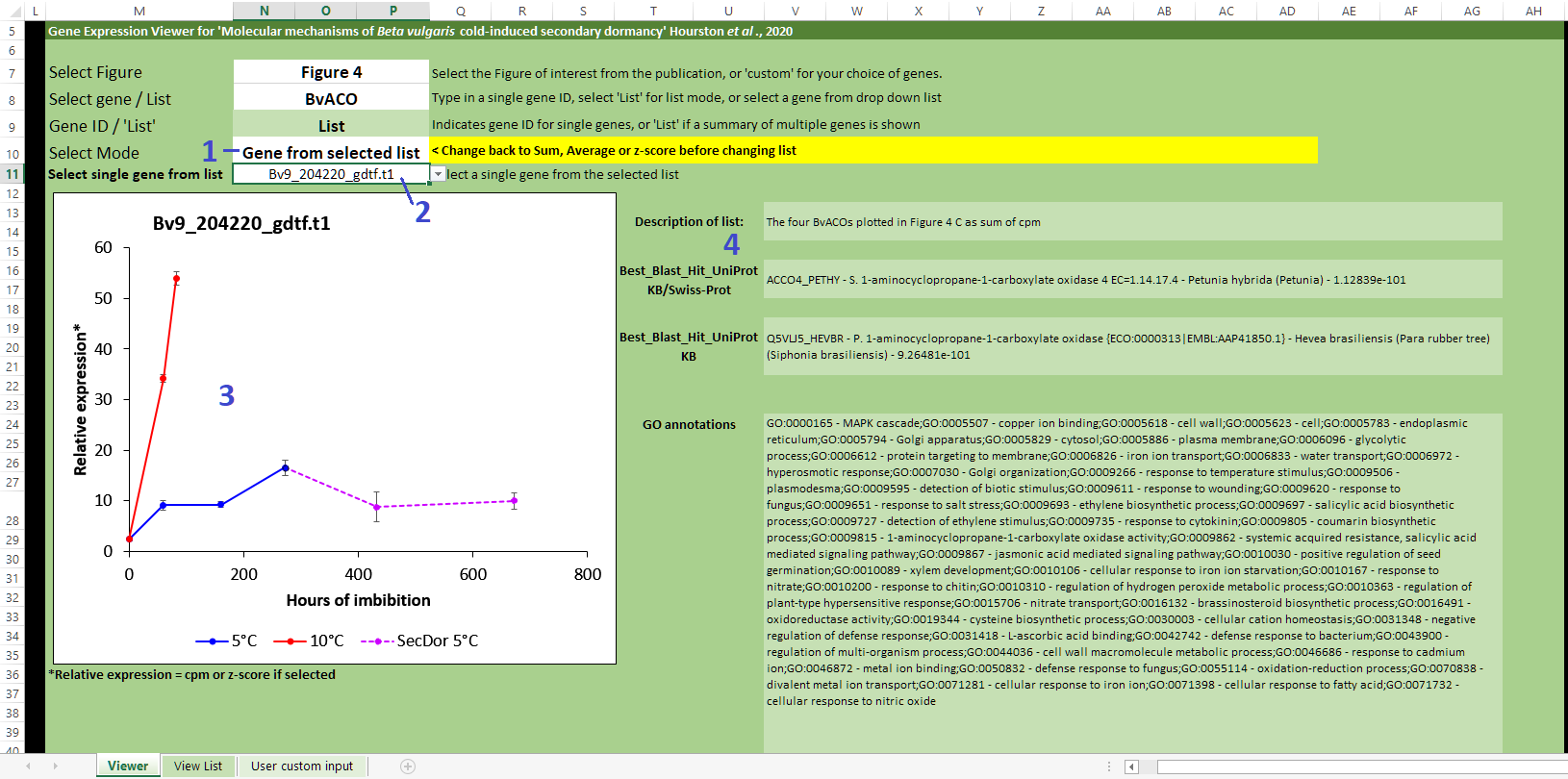


To view plots of the individual genes that make up the preset gene lists, for example in the *BvACO* list selected previously, select ‘Gene from selected list’ from the ‘Select Mode’ list (1), then select an individual gene from the ‘Select single gene from list’ drop down menu (2) (e.g. Bv9_204220_gdtf.t1). The graph will now display the mean cpm (± standard error) expression time-course for the experiment for this gene (3). The annotations for the selected gene will also be shown (4). Before changing the ‘Select gene / List’ or ‘Select Figure’ drop down menus please change ‘Select Mode’ to ‘Sum’, ‘Average’ or ‘z-score’ first (1).

***Viewing your own chosen single genes***

**
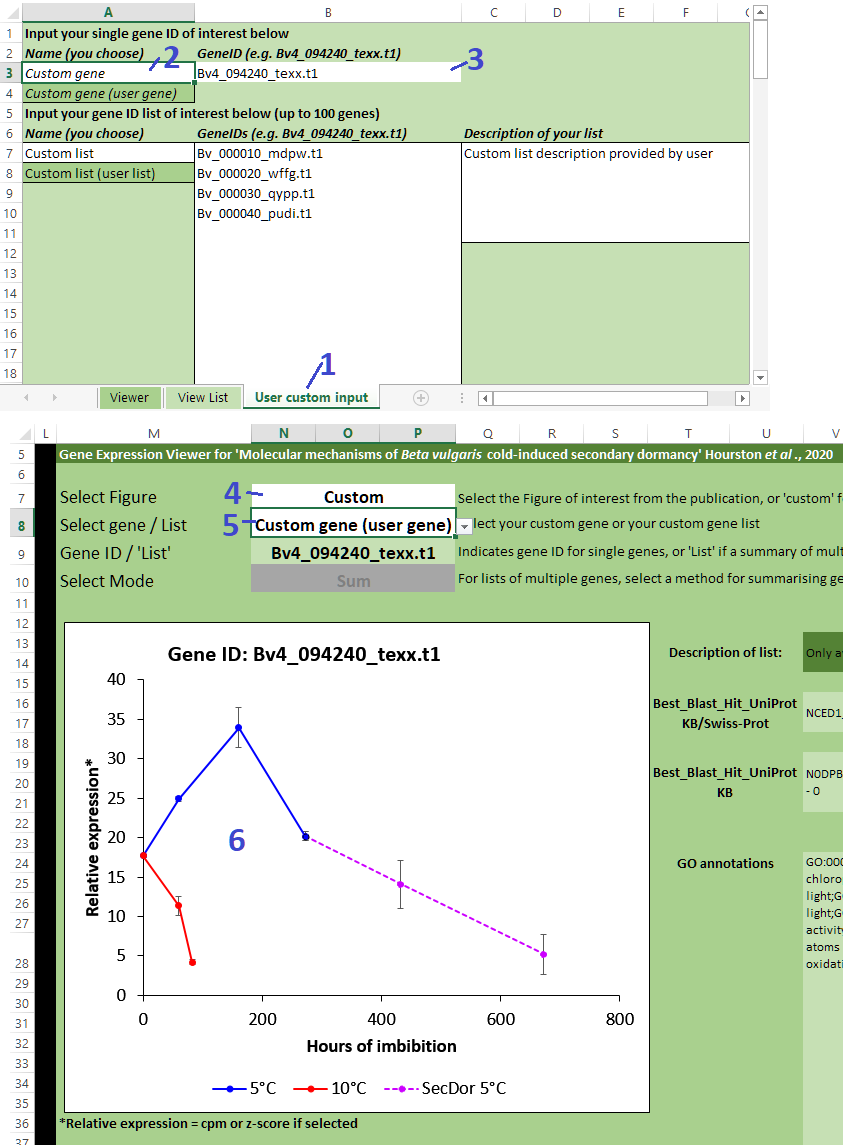
**

To view a gene of your choice go to the ‘User custom input’ sheet (1). Go to your custom gene name (optional) (2) and chosen gene ID (3). Return to the ‘Viewer’ sheet and select ‘Custom’ from ‘Select Figure’ (4) then ‘Custom gene (user gene)’ (or ‘*your chosen name of gene* (user gene)’). The graph will now display the mean cpm (± standard error) expression time-course for the experiment for this gene (5).

***Viewing summaries of your own chosen gene lists***

**
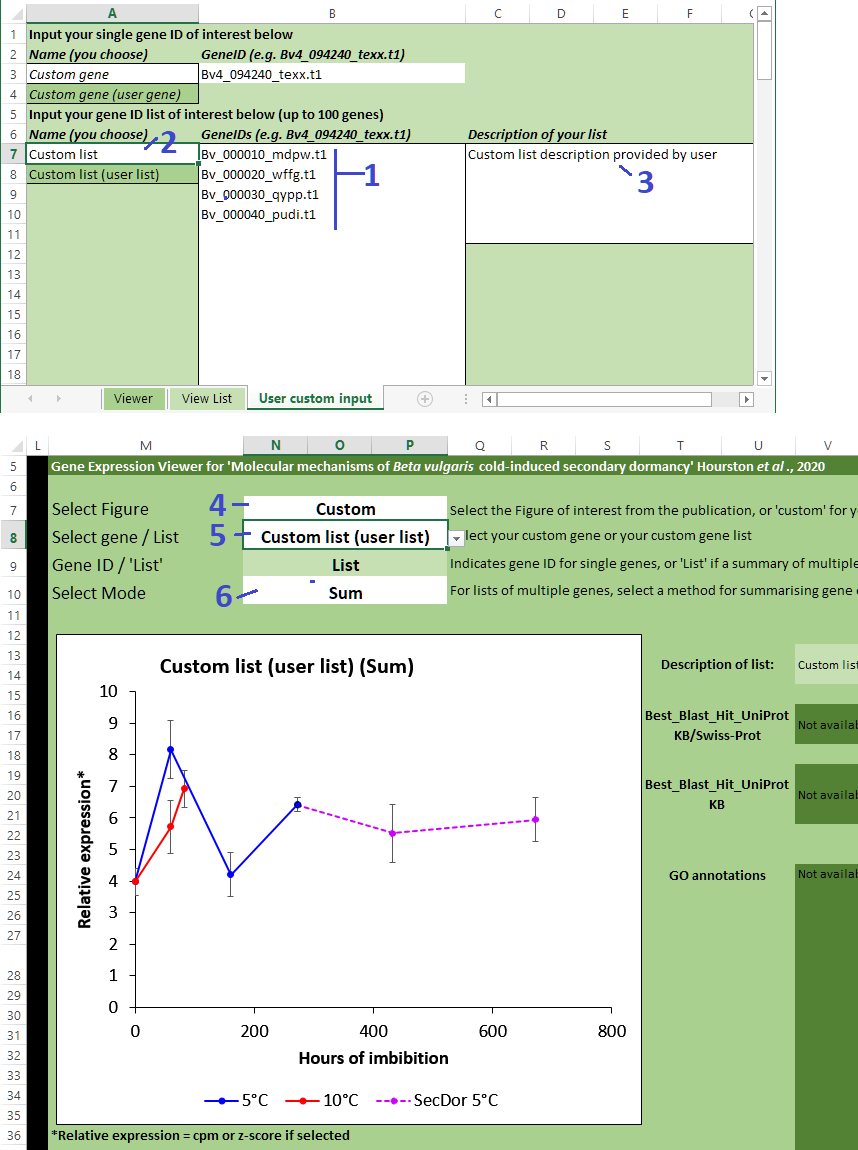
**

In the ‘User custom input’ sheet add up to 100 gene IDs to the GeneID list (1). You can optionally choose to add a name (2) and description (3) for your list. Return to the ‘Viewer’ sheet and select ‘Custom’ from the ‘Select Figure’ drop-down menu (4), select ‘Custom list (user list’) (or ‘*your chosen name of list* (user list)’) from the ‘Select gene / List’ drop-down menu (5). Then as above for the preset lists, select the summary mode (6) or view information about the individual genes in the list.
